# Triangulating evidence in health sciences with Annotated Semantic Queries

**DOI:** 10.1093/bioinformatics/btae519

**Published:** 2024-08-22

**Authors:** Yi Liu, Tom R Gaunt

**Affiliations:** 1 MRC Integrative Epidemiology Unit, Bristol Medical School, University of Bristol, Bristol, United Kingdom; 2 NIHR Bristol Biomedical Research Centre, University of Bristol, Bristol, United Kingdom

**Keywords:** Annotation, Data mining, information retrieval, knowledge representation, natural language processing, ontology

## Abstract

**Motivation:**

Integrating information from data sources representing different study designs has the potential to strengthen evidence in population health research. However, this concept of evidence “triangulation” presents a number of challenges for systematically identifying and integrating relevant information. These include the harmonization of heterogenous evidence with common semantic concepts and properties, as well as the priortization of the retrieved evidence for triangulation with the question of interest.

**Results:**

We present ASQ (Annotated Semantic Queries), a natural language query interface to the integrated biomedical entities and epidemiological evidence in EpiGraphDB, which enables users to extract “claims” from a piece of unstructured text, and then investigate the evidence that could either support, contradict the claims, or offer additional information to the query.

This approach has the potential to support the rapid review of preprints, grant applications, conference abstracts and articles submitted for peer review. ASQ implements strategies to harmonize biomedical entities in different taxonomies and evidence from different sources, to facilitate evidence triangulation and interpretation.

**Availability and Implementation:**

ASQ is openly available at https://asq.epigraphdb.org and its source code is available at https://github.com/mrcieu/epigraphdb-asq under GPL-3.0 license.

**Contact:**

yi6240.liu@bristol.ac.uk, tom.gaunt@bristol.ac.uk

## 1 Introduction

Researchers in health sciences are encouraged to seek multiple strands of complementary evidence to minimize the risk of bias creating false positives. This has been referred to as the *triangulation* (Lawlor et al. [2017]) of evidence, which may combine results from different study designs with different sources of bias (such as Sobczyk et al. [2023]), including from established findings in the literature (such as Vabistsevits et al. [2022]). One of the challenges raised by Sobczyk et al. [2023] regarding evidence triangulation is the harmonization of evidence, in terms of similarity of study subjects across study designs, common properties such as study population demography, as well as quality of evidence.

In addition, resource platforms which offer a portal to integrated heterogenous data such as Open Targets (Ochoa et al. [2021]) and EpiGraphDB (Liu et al. [2020]) are highly valuable sources which have the potential to support evidence triangulation by integrating evidence with relevant information from a range of dedicated data providers, including biomedical ontologies (Shefchek et al. [2020], Malone et al. [2010]), genetic associations (Elsworth et al. [2020]) and literature-derived evidence (Kilicoglu et al. [2012]). One of the main objectives for the web interface of such integrated data platforms is to present users with focused information from various integrated sources in order to facilitate the fast navigation and discovery of evidence. However, there is a need to improve accessibility of such complex data resources for less experienced users and to improve the interpretability of data, transforming source data into comprehensible evidence and knowledge regarding a research question. There are several challenges in order for these issues to be addressed, such as: how can a research question be represented so that evidence can be retrieved for triangulation, how should we integrate biomedical entities from different taxonomies and their relationships, and what strategies should we use to form larger *evidence* groups from heterogenous source data and methods to prioritize the retrieved evidence?

We approach the above-mentioned challenges by developing a scientific claim query platform Annotated Semantic Queries (ASQ; https://asq.epigraphdb.org) on top of the integrated biomedical knowledge and evidence of the EpiGraphDB (Liu et al. [2020]) platform, where users are able to investigate the various groups of evidence that support / contradict a claim, and further investigate the source data that relates to that claim. Scientific claims from a query text (e.g. the abstract of a preprint) are parsed as claim triples (in the form of a Subject PREDICATE Object *semantic triple*) in ASQ, where the subject and object terms are annotated with ontology terms and then mapped to evidence entities in an *entity harmonization* process, by a combination of high-dimensional text embeddings and sequence classification methods. Evidence from different sources are then retrieved and harmonized into two groups – a *triple and literature* evidence group composed of literature and literature-derived evidence, and an *association* evidence group composed of statistical association results from systematic analyses. Evidence items are then assigned scores which reflect their strength as well as their relevance to the claim, so that ASQ is able to present evidence which would be of potential high value to the user in order for them to assess and triangulate the evidence regarding a scientific claim. Here we discuss the implementation of the ASQ platform (Section 2) and the methods involved (Section 3), as well as demonstrate its use for systematic analysis of claims derived from medRxiv (Cold Spring Harbor Laboratory) submissions from 2020 to 2021 (Section 4). A glossary of terms is provided in Section S1 in the Supplementary Materials.

## 2 The EpiGraphDB-ASQ platform

**Fig. 1:**
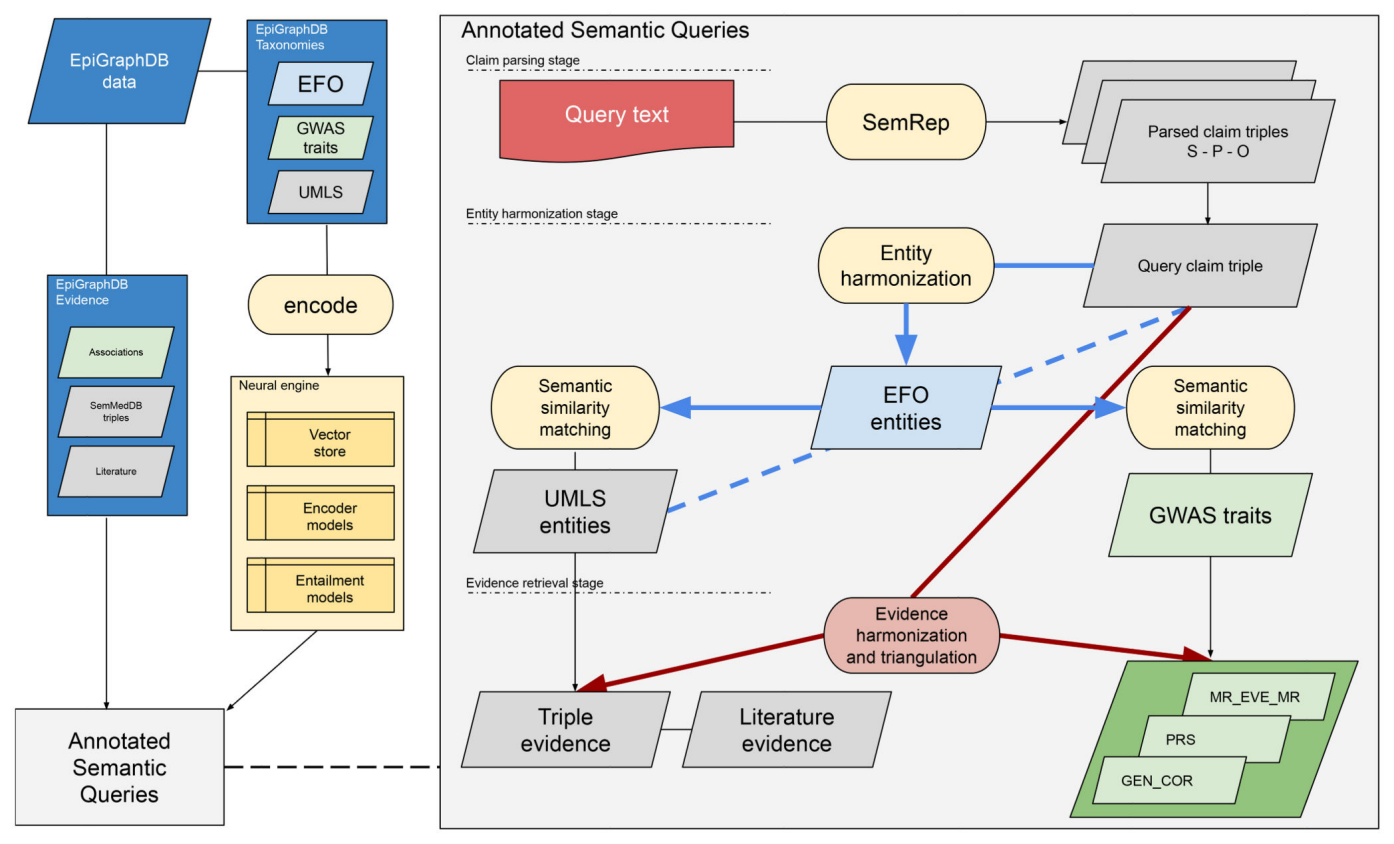
Architecture of the EpiGraphDB-ASQ platform Overall architecture design of the EpiGraphDB-ASQ platform and its associated components in the EpiGraphDB ecosystem. **Left**: EpiGraphDB’s biomedical entities (in the form of *graph nodes*) from different taxonomies are encoded into vector representations which allows for fast information retrieval against the query of interest. Epidemiological evidence (in the form of *graph edges*) are incorporated into ASQ as harmonized evidence groups. **Right**: Internal processing workflow of the EpiGraphDB-ASQ platform by the three stages: the claim parsing stage (Section 2), the entity harmonization stage (Section 3.1), and the evidence retrieval stage (Sections 3.2, 3.3, and S3).

The ASQ platform (Figure 1) is developed as a natural language interface to the epidemiological evidence integrated in EpiGraphDB database and ecosystem, with the aim of allowing users to access EpiGraphDB knowledge and triangulate the evidence using a simple scientific claim of interest as a starting point. For example, instead of relying on bespoke topic-specific web queries that are restricted to several entities or meta-entities or via structural queries to the database, ASQ presents the integrated evidence from EpiGraphDB as introspectable evidence items that “fact-check” either a claim (e.g. “Glucose TREATS Diabetes”) or a short piece of text by assessing such claims derived from the text.

On the web interface, the main entry point for a user to interact with the platform is to input paragraphs of scientific text (e.g. the abstract of a journal article or preprint). In this *claim parsing stage* we use SemRep (Kilicoglu et al. [2020]) as the query parser to derive **query claim triples** from the input text in the form of Subject-PREDICATE-Object (e.g. “Obesity CAUSES Asthma”), where the subject and object terms are part of Unified Medical Language System (UMLS) Metathesaurus (Schuyler et al. [1993]) and the predicate term is part of UMLS Semantic Network relationships. The user is then asked to select a specific triple of interest as the target of the downstream stages of entity harmonization and evidence retrieval. Alternatively, users can either directly input a query claim in the query triple view (https://asq.epigraphdb.org/triple), or start from the medRxiv systematic analysis summary results (https://asq.epigraphdb.org/medrxiv-analysis).

In the following *entity harmonization* stage (Section 3.1), ASQ harmonizes the biomedical entities from the claim triple with the Experimental Factor Ontology (EFO, Malone et al. [2010]) entities, with the EFO ontology serving as the float to connect the query entities and any evidence entities. By default ASQ attempts to retrieve entities that are semantically highly related (but not necessarily identical) to the query entities to allow for exploratory discovery about further evidence of potential interest. This can be adjusted to more specific or more sensitive mapping.

In the *evidence retrieval* stage, evidence items from EpiGraphDB are retrieved and harmonized into categories for evidence triangulation with the query claim based on the following dimensions:

- **Evidence groups** (Section 3.2) based on the source nature of the evidence item: (a) a *triple and literature* evidence group, or (b) an *association* evidence group.
- **Evidence types** (Section 3.3) based on the relationship of the evidence item with respect to the claim: (a) it supports the query claim (“supporting”), (b) it contradicts with the query claim with retrieved items indicating evidence in the opposite direction to the claim (“reversal”), (c) it fails to meet the required evidence threshold to be supporting or contradictory (“insufficient”) or (d) it could be of additional information (“additional”) to the claim.

Retrieved individual evidence item and groups of items are then measured and prioritized based on the following aspects: 1) the proximity of the involved entities in the evidence item, in terms of semantic similarity, to the entities of the query claim, as well as 2) the quantifiable strength of the evidence item (Section S3). Evidence scores for individual items and for groups will allow users to rapidly evaluate a wide range of evidence, whilst at the same time being able to assess the value of individual items and groups to the query.

**Fig. 2:**
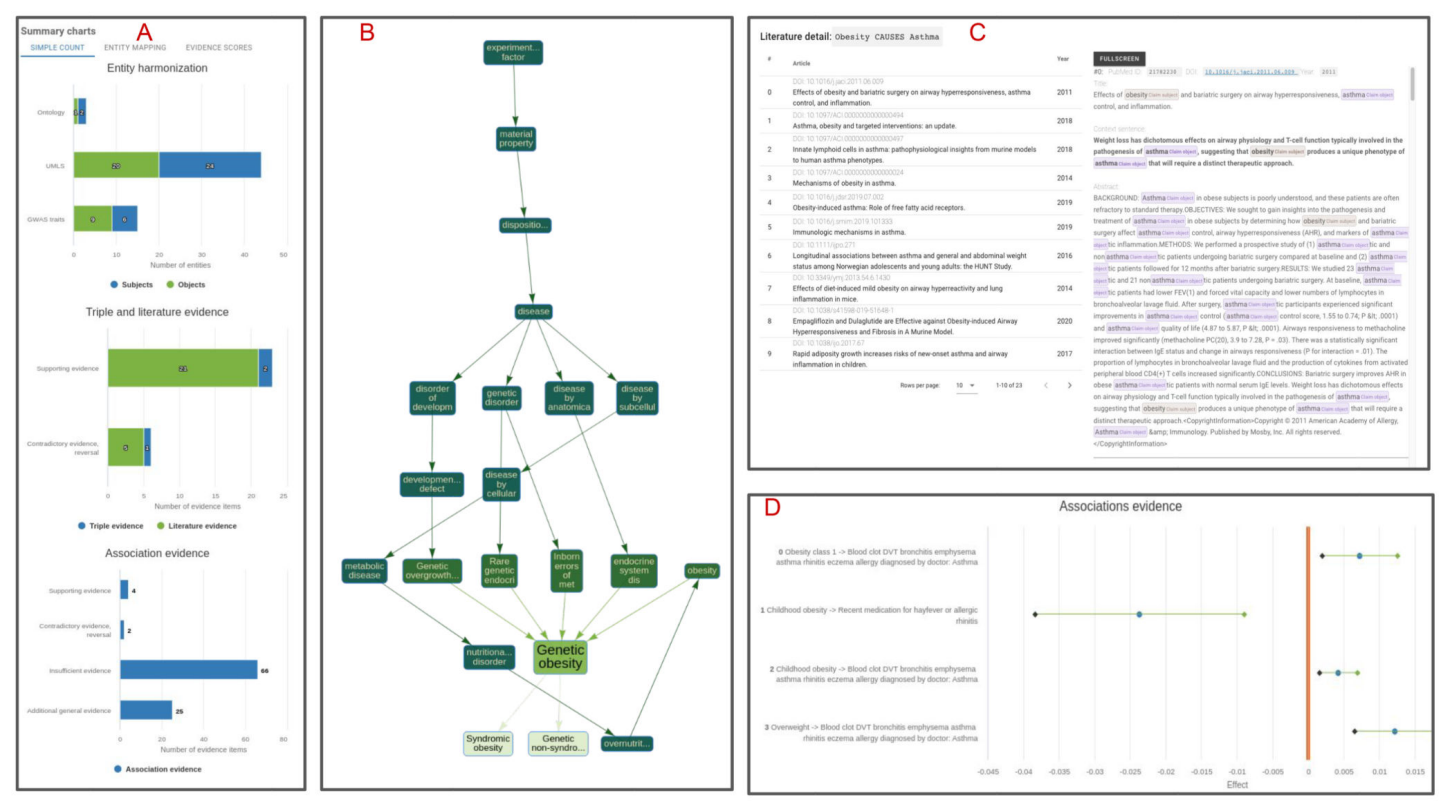
Overview of web interface Overview of the web interface functionalities of EpiGraphDB-ASQ. **A**: Summary of harmonized entities and retrieved evidence regarding the query claim. **B**: Sub-graph representation of a retrieved ontology entity in the EFO graph. **C**: Literature evidence: Summarized literature information (left) and context details (right) for a retrieved semantic triple “Obesity CAUSES Asthma”. **D**: Association evidence: Forest plot on the statistical association evidence regarding the query claim.

ASQ provides comprehensive information (Figure 2) regarding the status of entity harmonization with summary diagrams describing the mapped ontology entities and harmonization metrics linking the evidence entities to the original query claim. For triple and literature evidence, users can further introspect the context detail from which the semantic triples are derived, and for association evidence, ASQ displays the statistical results on forest plots as quantitative comparisons. In addition to the default interactive session on the web interface, ASQ offers programmatic access via the API (See “Code availability” section) which allows for batch processing and analysis (e.g. Section 4.1).

## 3 Methods

Here we discuss the methodological components in ASQ. Further details on the technical implementation of ASQ are also available in the Supplementary Materials: 1) further details on entity harmonization (Section S2), 2) score metrics to measure and prioritize retrieved evidence (Section S3), 3) improvement in entity and evidence retrieval based on semantic mapping (Section S4), and 4) further elaboration and explanation of included EpiGraphDB data (Tables S4, S5, S6, and S7).

### 3.1 Entity harmonization

Here we denote a **taxonomy** as a catalog of terms in a specific domain (i.e. a domain-specific vocabulary). For example, EpiGraphDB curates statistical association evidence between phenotypic traits which are denoted as trait names of genome-wide association studies (GWASes), as well as literature-derived semantic triple evidence from SemMedDB (Kilicoglu et al. [2012]) where subjects / objects of those triples are denoted as UMLS Metathesaurus terms. The GWAS traits and UMLS terms are thus separate *taxonomies* with domain-specific terminologies. An **entity** is then defined as a member of a *taxonomy*, i.e. a biomedical concept can be represented in a taxonomy as one of its predefined members with an identifier and a label (e.g. UMLS term C1305855 “Body mass index”). An **ontology** such as EFO where biomedical concepts are represented in a hierarchical tree can then be used to harmonize the various *entities* from different *taxonomies*, so that we are able to map the evidence *entities* curated in the knowledge graph (in this case EpiGraphDB) with those *entities* identified from the claim (i.e. subject and object terms from the claim triple). Conceptually we refer to this process as **entity harmonization**, as it harmonizes entities from different taxonomies into a unifying structure in the ontology. Our objective is to retrieve entities from EpiGraphDB that are semantically similar and ontologically meaningful with respect to the query terms, while ensuring broader relevant terms are retrieved by not restricting to identical token-level resemblance (which can also be achieved in ASQ by setting very high semantic similarity thresholds).

**Fig. 3:**
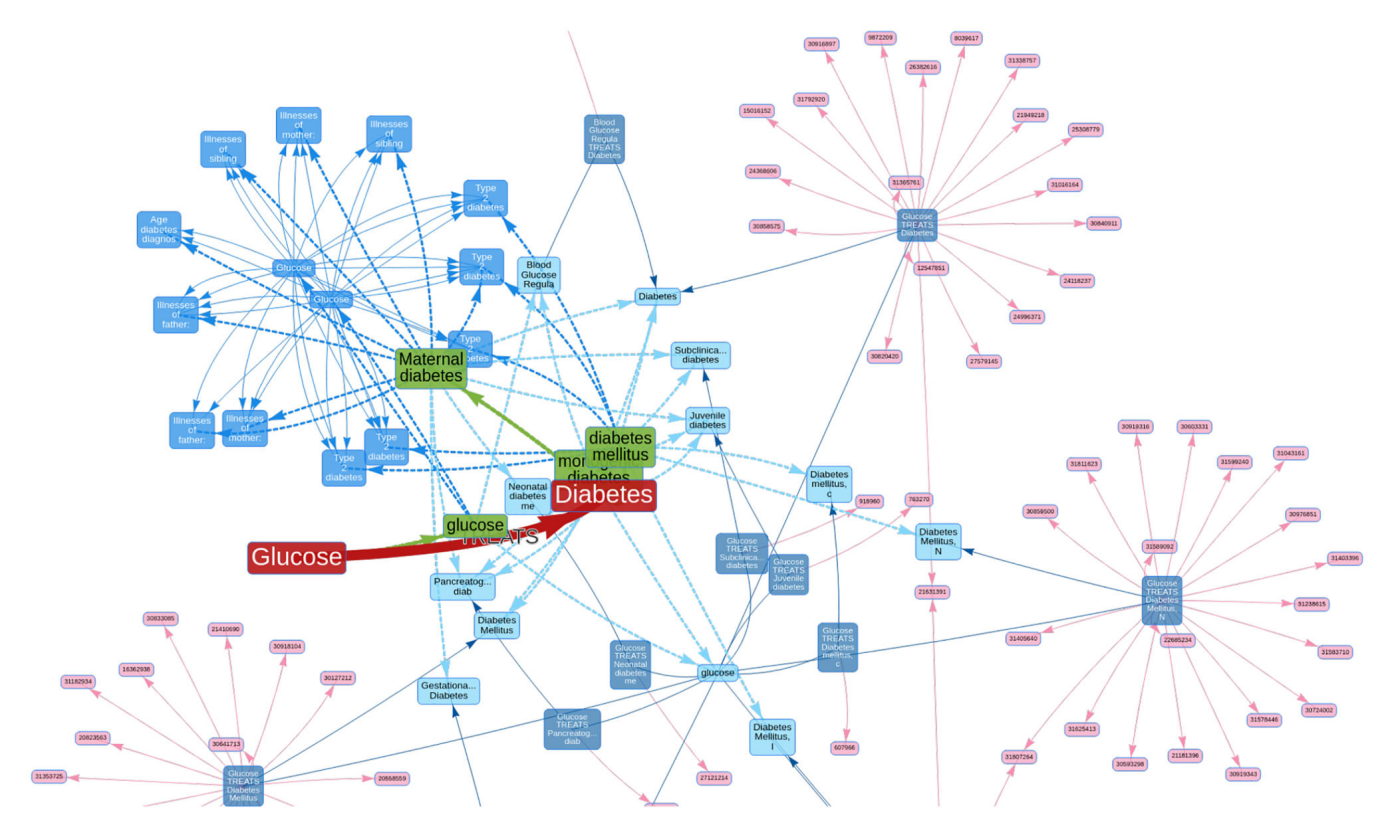
Evidence triangulation regarding scientific claims A summary network diagram on the retrieved entities and evidence from the ASQ platform regarding a claim “Glucose TREATS Diabetes”. The subject and object terms of the query claim are represented as nodes in red, and the predicate as a directed edge. The ontology term (green nodes) “glucose” is identified as the mapped term for the claim subject, and ontology terms “diabetes mellitus”, “monogenic diabetes”, “Maternal diabetes” are identified as the mapped terms for the claim object in the default setting (which can be adjusted at an interactive session or updated after initial results). Triple and literature evidence are represented as semantic triples (deep blue nodes) formed by UMLS Metathesaurus terms (light blue nodes), which are linked to source literature findings (pink nodes). For association evidence, statistical association results on GWAS traits (blue nodes) are represented as edges between them. Edges in dashed lines represent mappings between taxonomies, and edges in solid lines represent evidence items.

The entity harmonization stage process in ASQ is implemented as a two-stage approach (claim entities to ontology entities, then ontology entities to evidence entities) based on a mapping process automated by BlueBERT-EFO (Liu et al. [2023]) and ScispaCy (Neumann et al. [2019]). For a claim triple “Glucose TREATS Diabetes” (Figure 3) as an example, the claim subject term “Glucose” as a claim entity will first be mapped to its close ontological counterpart in EFO (i.e. CHEBI:17234 “glucose”) in the first stage, and in the second stage corresponding evidence entities from the *triple and literature* group and the *association* group (Section 3.2) i.e. a UMLS term C0017725 “glucose” and two “glucose” GWASes (ukb-d-30740_irnt and ukb-d-30740_raw from OpenGWAS (Elsworth et al. [2020])) are then further retrieved from ASQ. Similarly, for the claim object term “Diabetes”, its corresponding terms from the ontology and then from the evidence groups are retrieved based on their semantic similarities. We further discuss the technical details of this process in Section S2.

### 3.2 Evidence groups: retrieval of EpiGraphDB evidence

Various data components from EpiGraphDB are incorporated into the ASQ platform as two **evidence groups** below. Table 1 reports the further breakdown of evidence items, and further information regarding EpiGraphDB data sources are available in Table S7.

1. A *triple and literature* evidence group which consists of 1) semantic triples derived from literature sources that are curated from SemMedDB, and 2) the source literature articles from PubMed from which these triples are derived (Tables S4 and S5). Typically an evidence item in this group is composed of a semantic triple in the form of Subject-PREDICATE-Object (e.g. “Obesity CAUSES Asthma”) and the multiple source literature items containing specific context details in the literature title and abstract text.
2. An *association* evidence group which consists of various sources of curated systematic statistical association analysis studies between two GWASes (Table S6): 1) MR-EvE systematic Mendelian randomization analyses (Hemani et al. [2017]), 2) genetic correlations (Abbot et al. [2020]), and 3) polygenic risk score associations (Richardson et al. [2019]). ASQ incorporates the common properties of *effect size, standard error, P-Value*, as well as *source/target* GWAS traits from the source analysis data as the common quantitative/qualitative information of the evidence items, and additional detailed source-specific properties are also retrieved for users’ own investigation.

### 3.3 Predicate groups and evidence types: how retrieved evidence relate to the claim

Another dimension in the harmonization of evidence is the relationship of retrieved evidence to the claim, i.e. if it supports the claim and at what quantifiable level. We categorize claim triples into two **predicate groups** below according to the direction of the predicate. The set of included predicates is determined by the availability of corresponding predicates in the (LiteratureTriple) component of EpiGraphDB (see Section 2 and Table 1).

- *Directional* claims involving UMLS relationships “AFFECTS”, “CAUSES”, “PRODUCES” and “TREATS” as their predicates
- *Non-directional* claims involving UMLS relationships “ASSOCIATED_WITH”, “COEXISTS_WITH”, and “INTERACTS_WITH” as their predicates.

The **evidence types** for each of the **evidence groups** and **predicate groups** are defined as below and summarized in Table 2.

- *Supporting* evidence items are those that provide sufficient evidence in support of the query claim. For *triple and literature* evidence, evidence from mapped literature terms which share the same predicate with the claim triple are *supporting* evidence. For *association* evidence, when the claim is *directional*, evidence items from directional associations (e.g. a causal Mendelian randomization estimate) with the same direction of the claim will be candidates for supporting, whereas when the claim is *non-directional*, evidence items from all association sources will be candidates. In addition, potential candidate evidence items would also need to have sufficient evidence strength (Section S3) to qualify as valid evidence items in the *supporting* group.
- *Reversal* evidence items are those that would sufficiently contradict the claim with identified evidence from the reverse direction, and therefore is only applicable to *directional* predicates. In other words for a claim triple “Obesity CAUSES Asthma”, an evidence item that would *support* a counter triple “Asthma CAUSES Obesity” would be considered a *reversal* evidence item to the claim triple as it is in the opposite direction.
- *Insufficient* evidence items are those potential supporting evidence and reversal evidence items (when applicable) which fail to meet the desired strength of evidence. This only applies to *association* evidence, where we use P-Value as the quantitative measure. The aim of identifying *insufficient* evidence is to provide findings on the existence of systematic results, i.e. to determine whether the lack of evidence for a claim of interest is due to the absence of evidence (e.g. not curated by EpiGraphDB), or due to existing results failing to support/contradict a claim with sufficient strength.
- *Additional* evidence items are identified as evidence that could be of potential interest to users for further investigation, but which may not be sufficiently specific to inform the acceptance or rejection of a claim. For *association* evidence, when the claim is *directional*, non-directional evidence items are then candidates for *additional* evidence.

## 4 Results

### 4.1 Systematic analysis of medRxiv submissions

#### 4.1.1 Study design

**Fig. 4:**
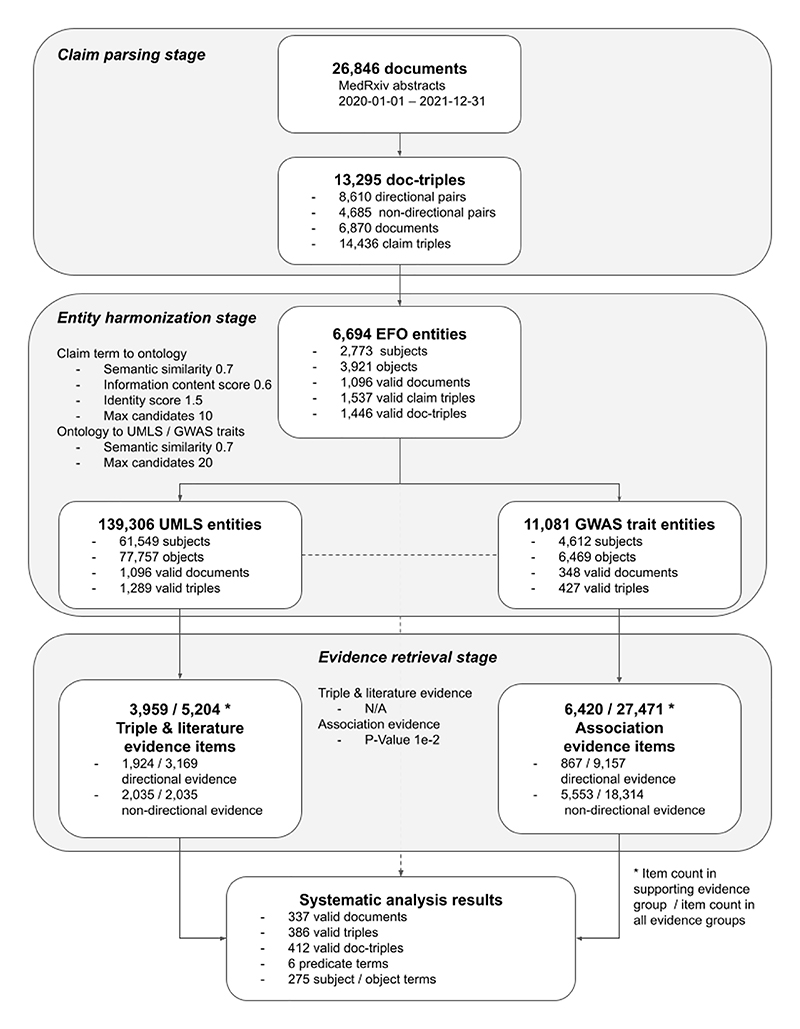
Study design of the systematic analysis and result metrics Overview diagram on the systematic analysis results and the primary metrics in the various stages discussed in Section 4.1. This figure complements Figure 1 regarding an individual case with the aspect of systematic scale. Further discussions on the parameter configuration as shown in each of the stages are available in Table S8.

We demonstrate the use of ASQ by systematically analyzing the preprint submissions on medRxiv in the sample period from 2020-01-01 to 2021-12-31 (Figure 4). Using the medRxiv/bioRxiv API, we identified 28,846 unique submissions in the period (in the case of multiple versions in a submission we kept only the initial version) and retrieved their abstracts as candidate text documents containing multiple scientific claims to be parsed in SemRep. Out of all the candidate documents, 13,999 documents were successfully parsed by SemRep to contain coherent semantic triples at sentence level, and 6,870 documents were identified to contain suitable predicates in ASQ’s *predicate groups*. In total we extracted 13,295 document-triples (14,436 claim triples) as the sample dataset.

Each claim triple was processed through ASQ programmatically to map with EFO and evidence entities in the entity harmonization stage using a set of parameters which are equivalent to the default settings used in the web interface (see Table S8 for configuration of parameters), with 1,446 document-triples identified to be valid and associated with entities in EpiGraphDB. Among these document-triples, we found that “Disease or Syndrome” is the most numerous semantic group (888 query terms, 7,831 EFO entities), followed by “Mental or Behavioral Dysfunction” (125 query terms, 1,708 EFO entities), and “Neoplastic Process” (138 query terms, 1,516 EFO entities; Table S10). In order to avoid the document-triple dataset for analysis being too large we used the intersection subset where a document-triple must contain at least one type of evidence in both the *triple and literature evidence* group and the *association evidence* group (Table S9 reports the summary statistics). The final dataset consisted of 412 claim triples (337 submission abstracts, and 386 document-triples) where each claim is associated with retrieved evidence from multiple evidence types across the evidence groups, and can be accessed via https://asq.epigraphdb.org/medrxiv-analysis with the option to adjust settings for individual query cases.

#### 4.1.2 Systematic results from entity harmonization and evidence retrieval

**Fig. 5:**
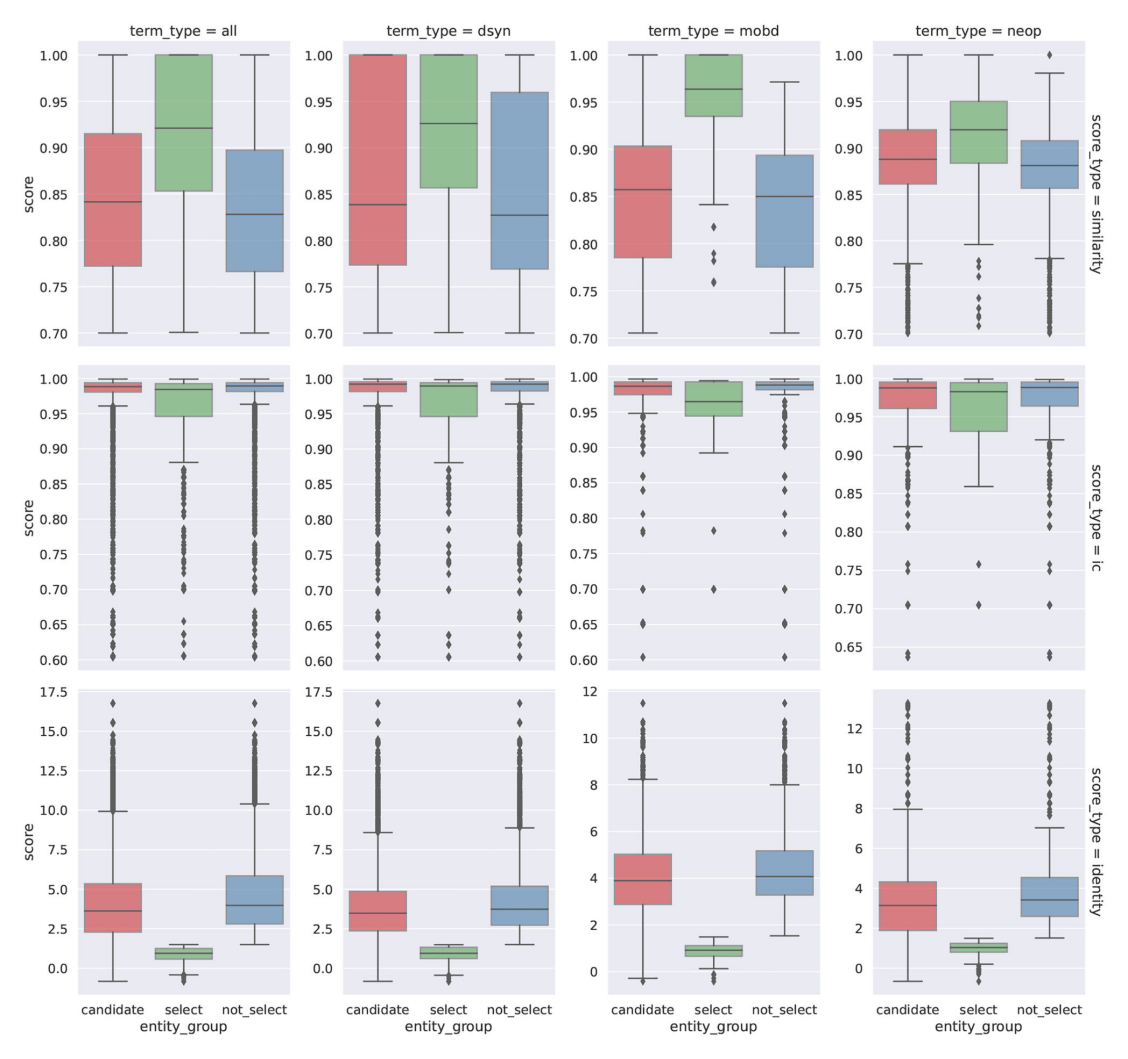
Entity harmonization stage: distribution of score metrics of retrieved EFO entities Distribution of semantic similarity scores, information content scores, and identity scores for retrieved EFO entities in the process of mapping with query UMLS terms, categorized by the semantic type of the UMLS term (“term_type”) and score metrics (“score_type”) in the retrieval process. **Category type**: “candidate” for entities retrieved as a candidate, “select” for candidates that are selected in the automated process (Section 3.1), and “not_select” for candidates that are not selected. **Left** (“all”): Distribution across all semantic types. **Middle 1** (“dsyn”): In the “Disease or Syndrome” group. **Middle 2** (“mobd”): In the “Mental or Behavioral Dysfunction” group. **Right** (“neop”): In the “Neoplastic Process” group. This figure reports distributions in the top 3 semantic type groups by entity count (Table S10 reports entity counts of all semantic types). **Top** (“similarity”): similarity of term By semantic similarity score to measure embedding vectors. **Center** (“ic”): By information content score to measure the concreteness of the term in EFO. **Bottom** (“identity”): By identity score to measure the inferred relative distance of the UMLS term. The roles of the score metrics take in the harmonization retrieval process are discussed in detail in Section 3.1. Figure S3 reports the distribution of score metrics for retrieved UMLS and trait entities.

In the entity harmonization stage of the systematic analysis, the retrieval of EFO entities is determined by an initial stage where EFO candidates are retrieved by the semantic similarities between the EFO candidates and the query subject/object terms by their encoded text vectors, and a subset is subsequently selected based on proximity of the query term and the candidates in the EFO graph as indicated by the identity scores, which then gets mapped to evidence entities via semantic similarities (See Section 3.1). Figure 5 shows the distribution of score metrics for the entity harmonization process where query terms are mapped to EFO entities and Figure S3 shows the distribution of scores for mappings of evidence entities to the original query terms. For selected entities which have identity scores below the threshold in the automated process, they would also be semantically closer to the query terms than the rest of the retrieved candidates (with mean semantic similarity scores above 0.9), and therefore from a systematic scale ASQ is able to select a set of corresponding EFO entities that have high association to the query terms of interest as the basis for further retrieving evidence entities related to these query terms. A similar automated approach applies in the interactive session, and users are able to optionally override the automated processing of entity harmonization with manual selection of EFO entities of interest or re-adjust the entity selection afterwards.

**Fig. 6:**
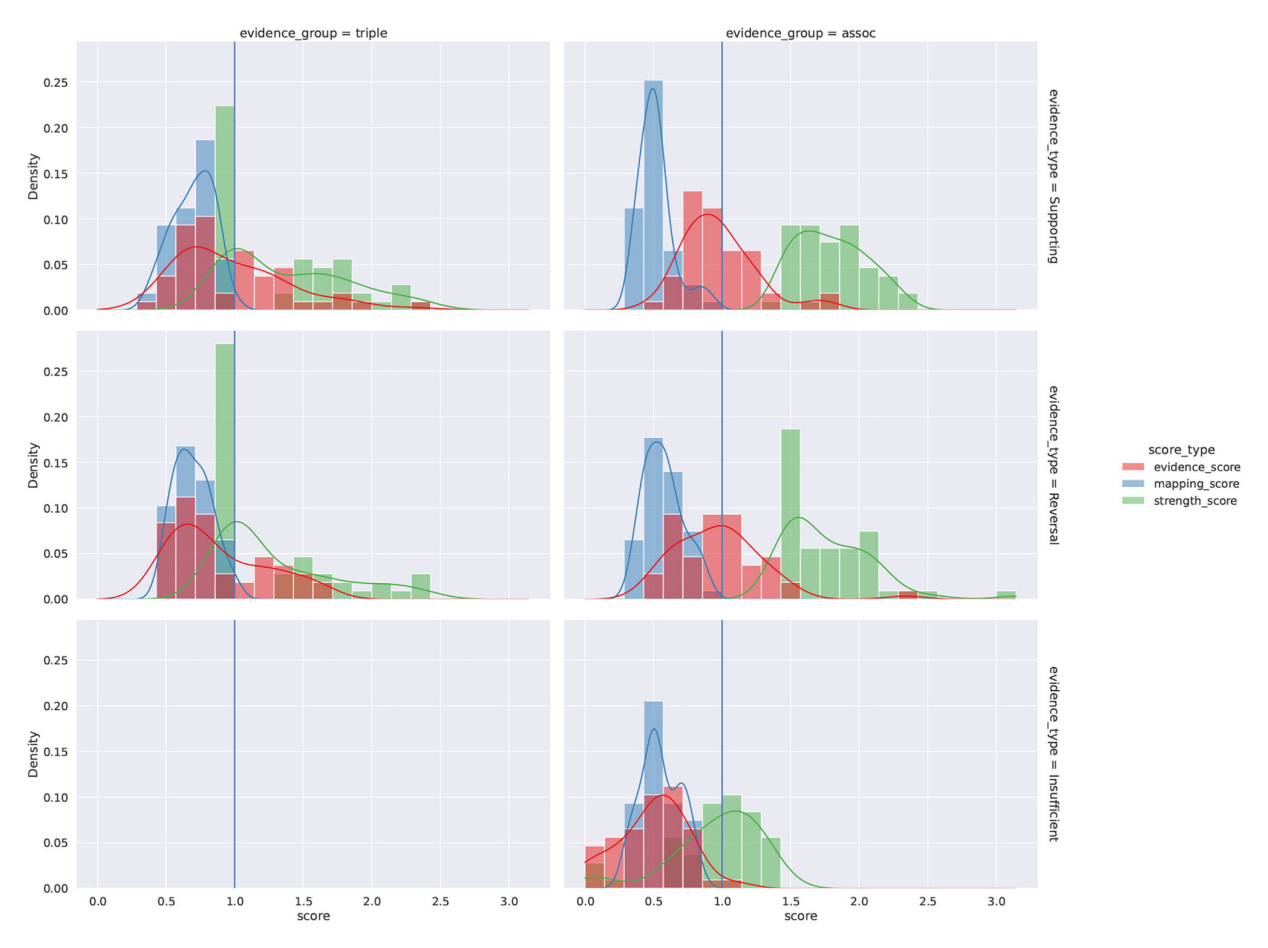
Evidence retrieval stage: distribution of evidence scores and constituent scores Distribution of evidence scores and its constituent scores (entity mapping scores and evidence strength scores), for the “supporting”, “reversal”, and “insufficient” evidence types (by rows) in the triple and literature evidence group and the association evidence group (by columns). This figure reports aggregated distributions across directional and non-directional predicate groups, and Tables S6 and S7 report detailed distributions by evidence groups, evidence types, and predicate groups. Note an “insufficient” evidence type is only applicable to the association (“assoc”) evidence group and not the triple and literature (“triple”) evidence group.

Figure 6 shows the distribution of the evidence scores and their constituent scores in the evidence retrieval process, and further details are available in the Supplementary Materials on group specific distributions (Figures S6 and S7), as well as on summary statistics (Table S11). The mean mapping score for retrieved evidence is around 0.5 to 0.7, with a typical scenario where the retrieved entities have about 0.85 to 0.92 in semantic similarity to its upstream entities. The mean strength score for *triple and literature evidence* is around 1, i.e. on average a triple evidence item was identified in only 1 source literature item, though there is a long upper tail with many cases of 10 to 20 source literature items associated with a triple (i.e. strength scores around 2 to 2.3). Association evidence items are required to have sufficient strength of effect size in order to qualify into the “supporting” type or the “reversal” type (otherwise they would be of “insufficient” evidence), and therefore the strength scores for “supporting” and “reversal” types are markedly higher than items in the “insufficient” type. In general, the evidence scores for “supporting” and “reversal” *association evidence* are found to be distributed around the baseline score of 1. In addition, as constituent scores, the mapping scores and strength scores contribute to the evidence scores in roughly linear relationships (Figures S4 and S5).

#### 4.1.3 Top cases of research areas

**Fig. 7:**
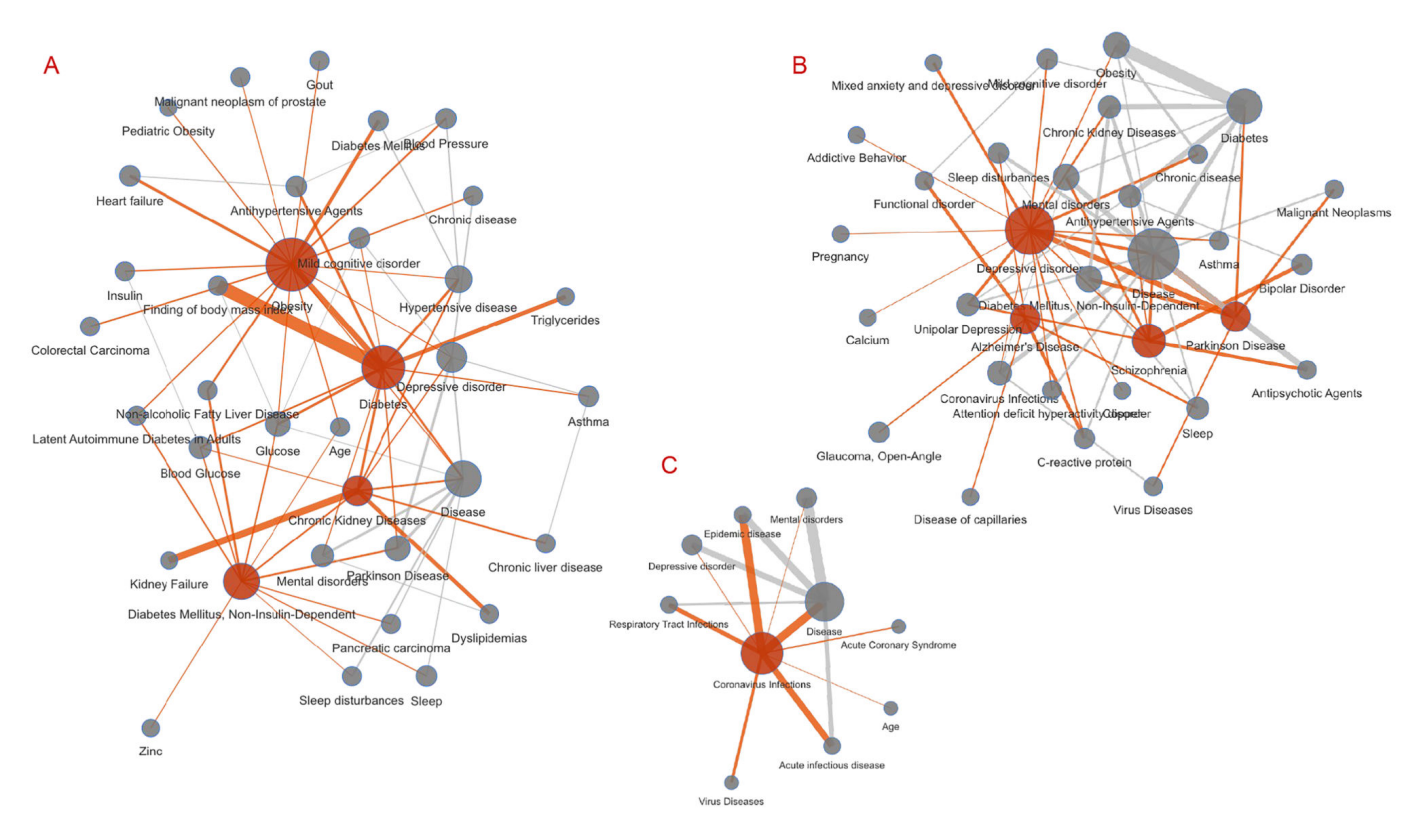
Systematic analysis results: evidence clusters of topic terms Clusters representing research interests as parsed from the MedRxiv abstract sample from 2020-01-01 to 2021-12-31 as well as their corresponding evidence retrieved from EpiGraphDB-ASQ as network diagrams. Nodes colored in red correspond to the primary claim terms (Table 3) and edges colored in red correspond to relationships involving a primary claim term. **A**: Obesity cluster with primary terms “Obesity”, “Diabetes”, “Diabetes Mellitus, Non-Insulin-Dependent”, “Chronic Kidney Diseases”; **B**: Mental illness cluster with primary terms “Depressive disorder”, “Alzheimer’s Disease”, “Schizophrenia”, “Parkinson Disease”; **C**: COVID-19 cluster with primary terms “Coronavirus infections”; The diagrams are generated by retrieving first-degree neighbor nodes for each of the top term nodes, where node size corresponds to term count, and edge width correspond to aggregated supporting evidence scores between nodes. Interactive diagram is available on https://asq.epigraphdb.org/medrxiv-analysis.

Whilst results from the systematic analysis reflect the availability of evidence in ASQ and EpiGraphDB in various areas, they also show the popular research topics and themes reflected from medRxiv submissions in 2020-2021. Figure 7 shows several clusters of research areas with central terms as measured in Table 3, and example claim triples can be found in Table S12. In addition to research associated with the COVID-19 pandemic (“Coronavirus infections”), the two areas with highest research submissions and retrieved evidence are regarding obesity and associated diseases (“Obesity”, “Diabetes”, “Diabetes Mellitus, Non-Insulin-Dependent”, “Chronic Kidney Diseases”, etc.) and mental health (“Depressive disorder”, “Parkinson Disease”, “Alzheimer’s Disease”, “Schizophrenia”, etc.). Notably when SemRep fails to recognize a more specific term it will fall back to more general terms, and therefore the term “Disease” is prominent in the list of top terms. Examples in Table S12 suggest claims involving predicates “CAUSES”, “AFFECTS” and “COEXISTS_WITH” are the most popular claims that can be derived from submitted abstracts, which is similar to the summarized results in all cases in Table S11 where these predicates are the ones with most retrieved evidence items.

#### 4.1.4 Study design

Here we showcase an example in demonstrating the use of ASQ for researchers in triangulating evidence regarding epidemiological research questions. From the systematic dataset, ASQ extracted a claim triple “Obesity CAUSES Heart failure” from a preprint abstract regarding a Mendelian randomization analysis investigating causal relationships between body mass index and heart failure risk (Lumbers et al. [2020]), derived from the context “About 40% of the excess risk of HF due to adiposity is driven by SBP, AF, DM and CHD”, where “HF” is recognized as heart failure and “adiposity” as obesity. These results can be found on ASQ (https://asq.epigraphdb.org/triple?subject=Obesity&object=Heart%20failure&predicate=CAUSES&analysis). The query subject “Obesity” and object “Heart failure” were mapped to their corresponding ontology counterparts then to evidence entities, from which ASQ then identified suitable evidence items. At aggregate level for *triple and literature evidence* there are more supporting evidence items (11) with higher aggregated scores (12.80) compared to reversal evidence items (6) with lower aggregated scores (5.46), similarly there are 5 supporting *association evidence* items with an aggregated score of 5.11 with no reversal evidence identified. Users are able to further investigate the literature that either associate with the claim triple (e.g. Sardu et al. [2019] and Baena-Díez et al. [2010]) or the reversal claim that heart failure might cause obesity (e.g. Goncalves et al. [2017]), viewing the surrounding context from the abstract directly in the ASQ interface, or clicking a link to access the original paper. For *association evidence* ASQ identified several individual findings from the pairwise Mendelian randomization studies with sufficient statistical significance as supporting evidence. ASQ also identified a range of findings that are insufficient in statistical significance to qualify as supporting evidence, which are useful both in showing the scope of evidence identification but also in determining the cause of a lack of reversal evidence. In this case, the lack of reversal evidence was due to absence of results from the MR-EvE data source. In addition, ASQ identified several findings from the polygenic risk score associations source, and since the identified trait term “Target heart rate achieved” was not directly equivalent to the query object “Heart failure” ASQ would assign low evidence scores to these findings in the context of the original claim.

In general ASQ is able to assist researchers in investigating research questions in epidemiology both at aggregate level to have an overview of the evidence categories regarding the question, as well as at individual level for researchers to further investigate the evidence items in literature or statistical findings with their expert knowledge.

## 5 Discussions

We developed the Annotated Semantic Queries (ASQ) platform both as an approach to improve the accessibility of the EpiGraphDB data and ecosystem for users through the implementation of an openly accessible natural language interface (whilst also enhancing programmatic access), as well as an analytical framework regarding the harmonization of biomedical entities and evidence for the purpose of triangulation on biomedical research questions. There is an intrinsic problem with integrated data platforms containing rich and complex data: experienced users wish to be presented with flexible access to the data in order to navigate to the elements they want, yet new users can find this complexity overwhelming (even if well documented). From this perspective ASQ provides an accessible natural language query interface for such users to find the evidence relating to a specific claim/question e.g. “Can obesity cause asthma?”, which can either be parsed from a short piece of text containing scientific claims, or directly input as a claim triple of Subject PREDICATE Object. In addition to providing a more accessible interface to EpiGraphDB this approach provides a novel way to systematically evaluate a piece of text (such as a preprint abstract) to identify whether claims within that text are supported by other data. Heterogenous knowledge types are harmonized in ASQ into intuitive evidence groups making triangulation of evidence in different groups more accessible, without either the need to navigate to various area-specific topics or the need to formulate complex queries. As we have demonstrated with our systematic analysis, the evidence retrieved by ASQ can be of high value and relevance to a wide range of researchers in epidemiology and health sciences, to assist the triangulation of evidence in their research. This is a generalizable approach that could be applied to a wider array of knowledge graphs and evidence sources to support the development of tools for rapid “semi-automated” (assisted) review of preprint.

As ASQ aims to provide accessible interfacing to heterogenous resources, it shares similar elements with previously reported semi-automated “fact-checkers” (or evidence assessment tools) for scientific findings such as works based on curated medical topics including on drug abuse (Cameron et al. [2013]), on vaccination attitudes and misinformation (Jitsuvax https://jitsuvax.github.io/) and on nucleotide sequence reagents (Labbé et al. [2019]). The value of ASQ is in providing a more general evidence assessment tool (based on a biomedical and epidemiological knowledge graph), and an easy to use web interface. Our work also relates to works on claim verifications involving the use of language models which are trained with curated evidence corpora (e.g. Wadden et al. [2021], Glockner et al. [2022], Wührl et al. [2023]) for “fact-checking” of the claim, whereas our use of language model is on the mapping of knowledge graph entities (*entity harmonization*) and use a more schematic rule based approach for the presentation of evidence with respect to the claims of interest (*evidence harmonization*). As the purpose of ASQ is assessing claims with evidence from a knowledge graph, our methods are related to other approaches such as machine learning models in Knowledge Graph Question Answering i.e. via graph embedding methods e.g. TransE (Bordes et al. [2013]) or RESCAL (Nickel et al. [2011]), as well as graph models based on attention-based Transformers (e.g. Ahmad et al. [2020]) or Large Language Models (LLM, e.g. Zhang et al. [2023a] and Banerjee [2023]). Lastly, our works relates to research involving the use of ontologies such as EFO and UMLS as basis of search queries (e.g. Mukherjea et al. [2005], Fu et al. [2012], Sarica et al. [2019]), and we contribute to this area of research from the perspective of using ontologies as basis of *evidence triangulation* across a knowledge graph.

ASQ applies our previous method development (Liu et al. [2023]) in combining sequence classification Transformer models with text vector embeddings for the harmonization of entities in different taxonomies. ASQ is able to combine the functionalities of parsing free text to generate structural claims with the harmonization of heterogenous entities and evidence to enable claims to be mapped to with evidence both from literature and semantic knowledge as well as evidence from systematic association analysis. This approach allows for sufficient interpretability at the various steps of evidence retrieval and prioritization to avoid the risk of “model hallucination” (Zhang et al. [2023b]) which is often a non-trivial issue for Large Language Models in language understanding and reasoning. In our future research we aim to incorporate more automated approaches (such as our previous study on graph embedding models, Lloyd et al. [2022]) into evidence triangulation, whilst maintaining a sufficient level of structural and introspectable retrieval and harmonization of evidence.

As part of the ASQ platform we developed a scoring mechanism to prioritize the retrieved evidence items, accounting for the semantic relevance of entities to the query of interest, as well as the strength of the evidence item per se. This score enables users to rapidly evaluate a wide range of evidence, whilst at the same time being able to assess the value of individual evidence items or evidence groups to the query to enable prioritization. On the other hand, it is worth pointing out that (as with other data platforms) users should not rely on metrics (whether they are ranking metrics, P-Values, or discrete categories of “accepting” / “inconclusive” / “rejecting”) as sole criteria when assessing evidence or as a substitute for detailed investigation. The nature of the heterogenous source data means we strongly recommend users spend time investigating individual evidence sources using links provided by ASQ, as well as understanding the various harmonization strategies in ASQ’s documentation, since data harmonization in itself is an opinionated way of data retrieval which might not be aligned with individual use cases. We seek to continue the development of ASQ in various aspects to improve the robustness of evidence retrieval and entity harmonization, as well as accessibility of evidence triangulation for researchers.

Whilst the ASQ platform offers an accessible approach to querying a knowledge graph and a novel way for evidence triangulation, there are some important limitations. The extraction of claims from a piece of text is rarely perfect, and genuine claims may be missed and others misinterpreted. The literature knowledge base represented by SemMedDB is also subject to the same limitations (using the same tool: SemRep), the triples extracted by SemRep are not context-specific (i.e. there is no information about which section of an abstract these come from, so may reflect hypothesis rather than conclusions) and in addition, literature evidence is subject to publication bias. The low extraction performance of SemRep is also another limit for ASQ, as its initial version was developed two decades ago (Rindflesch and Fiszman [2003]) despite receiving continuous updates (Kilicoglu et al. [2020]) and extensions (Ming et al. [2024]). ASQ would require an triple extractor that is able to extract not only entities but also relationships between the entities, and to our best knowledge there isn’t a mature tool that is able to supersede SemRep. We think future related research based on ASQ could focus on a small scale case study where an LLM finetuned extractor should be expected to perform with better recall and robustness. Lastly, the association evidence in EpiGraphDB is constrained to a subset of published GWAS datasets in OpenGWAS, and may omit important entities relating to claims in a query text. The ASQ approach should therefore be considered as a support tool that aids evidence identification to assess a claim, but not a comprehensive “fact-checker”.

## Supplementary Material

Supplementary Materials

## Figures and Tables

**Table 1:** Integration of epidemiological evidence in EpiGraphDB Distribution of the EpiGraphDB *knowledge* triples which are the *source* evidence in this study, harmonized into the two evidence categories. Column “Triples” and column “Literature” report respectively the number of literature triples and number of associated source literature articles in a triple and literature evidence group, and column “Associations” report the number of statistical associations in an association evidence group. For example, there are 37,423 literature triples in the form of Term 1 AFFECTS Term 2 where Term 1 and Term 2 are from the term types of aapp (“Amino Acid, Peptide, or Protein”), dsyn (“Disease or Syndrome”), gngm (“Gene or Genome”) (a UMLS term can have multiple associated types), and there are 57,928 source literature articles from which the 37,423 literature triples are derived. Similarly, there are 8,966,440 statistical associations from the MR-EvE study (Hemani et al. [2017]) between GWAS-es in the UKBiobank categories (ukb-a, ukb-b, etc.) of OpenGWAS (Elsworth et al. [2020]). A UMLS term can have multiple associated semantic types and the label descriptions on UMLS semantic types are available in Table S4.

Triple and literature evidence				
Direction	UMLS Predicate	UMLS term type	Triples	Literature
Directional	AFFECTS	aapp, dsyn, gngm	37,243	57,928
	AFFECTS	dsyn	29,167	58,753
	CAUSES	dsyn	85,231	222,462
	CAUSES	aapp, dsyn, gngm	49,178	100,681
	TREATS	phsu, dsyn, orch	82,263	274,589
	TREATS	phsu, dsyn	47,416	238,636
	PRODUCES	aapp, gngm	69,691	106,862
	PRODUCES	phsu, aapp, gngm	12,706	26,122
Non-directional	ASSOCIATED_WITH	aapp, dsyn, gngm	188,961	423,727
	ASSOCIATED_WITH	phsu, aapp, dsyn, gngm	29,425	86,176
	INTERACTS_WITH	aapp, gngm	393,759	673,470
	COEXISTS_WITH	aapp, gngm	224,098	332,834
	COEXISTS_WITH	dsyn	150,166	385,349
	INTERACTS_WITH	aapp, enzy, gngm	72,194	140,836
Association evidence				
Direction	Association type	GWAS categories	Associations	
Directional	MR_EVE_MR	ukb, ukb	8,966,440	
	MR_EVE_MR	prot, ukb	5,028,904	
	MR_EVE_MR	ubm, ukb	3,833,948	
	MR_EVE_MR	prot, prot	3,109,406	
	MR_EVE_MR	prot, ubm	1,974,611	
Non-directional	GEN_COR	ukb-b, ukb-b	453,752	
	GEN_COR	ukb-a, ukb-b	286,536	
	GEN_COR	ukb-a, ukb-a	180,536	
	GEN_COR	ukb-b, ukb-d	133,554	
	GEN_COR	ukb-a, ukb-d	84,266	
	GEN_COR	ukb-d, ukb-d	38,908	
	PRS	ieu-a, ukb-a	70,926	
	PRS	ukb-b, ieu-a	45,394	
	PRS	ukb-a, ukb-a	2,198	
	PRS	ukb-b, ukb-a	704	

**Table 2:** Classification of retrieved evidence Summary of how retrieved evidence items are classified based on the **predicate direction group, evidence group**, and **evidence type**. The notation *S* − *P* → *O* means a Subject PREDICATE Object triple where the predicate is directional (e.g. a “CAUSES” predicate versus a non-directional predicate “ASSOCIATED_WITH”) and the notation *S* − *P* − *O* means a triple with non-directional predicate.

	Supporting	Reversal	Insufficient	Additional
Directional predicates CAUSES, TREATS, PRODUCES, AFFECTS				
Triple and literature group	*S* − *P* → *O*	*O* − *P* → *S*	N/A	N/A
Association group	*S* − *P* → *O, P*_*P*_ − *Value* < *π*	*O* − *P* → *S, P*_*P*_ − *Value* < *π*	*S* − *P* → *O, P_P_* − *Value* ≥ π	non-directional *S* − *P* − *O*
Non-directional predicates INTERACTS. _WITH, COEXISTS_WITH, ASSOCIATED_WITH				
Triple and literature group	*S* − *P* − *O*	N/A	N/A	N/A
Association group	*S* − *P* − *O, P*_*P*_ − *Value* < *π*	N/A	*S* − *P* − *O, P*_*P*_ − *Value* ≥ *π*	N/A

**Table 3:** Systematic analysis results: top claim terms by retrieved evidence Top claim terms sorted by the number of cases where the query claim triple is associated with both triple and literature evidence as well as association evidence (“T&L. + Assoc.”). For example, there are 41 claim triples involving the term “Disease” as either a subject term or an object term where these claim triples are identified with *both* supporting evidence in triple and literature evidence (“T&L.”) and association evidence (“Assoc.”) groups, 74 cases identified with supporting triple evidence, 44 cases identified with supporting association evidence, 77 cases identified with any evidence types (“Any”; See Section 3.3 and Table 2 for all evidence types), and 715 cases from the triples in the initial claim parsing stage dataset (“Init.”; Figure 4) after parsing from source abstracts regardless of results in the downstream stages.

Claim term	Supporting			Any	Init.
	T&L. + Assoc.	T&L.	Assoc.		
Disease	41	74	44	77	715
Obesity	20	25	25	30	125
Diabetes	17	19	18	20	87
Depressive disorder	14	20	16	26	100
Parkinson Disease	13	13	13	13	111
Diabetes Mellitus, Non-Insulin-Dependent	10	12	12	15	84
Alzheimer’s Disease	8	10	8	10	111
Schizophrenia	8	11	8	11	32
C-reactive protein	7	7	9	10	24
Malignant Neoplasms	7	8	15	19	100
Chronic Kidney Diseases	6	9	6	9	35
Chronic disease	5	6	5	6	44
Fatigue	5	5	6	6	25
Sleep	5	5	6	6	21
Atrial Fibrillation	5	6	6	9	57
Pain	4	4	6	6	30
Glucose	4	5	4	6	20
Blood Glucose	4	5	4	5	15
Hypertensive disease	4	12	4	13	90
Mental disorders	4	8	4	10	42
Cardioembolic stroke	3	3	3	3	14
Testosterone	3	5	3	6	21
Diabetes Mellitus	3	4	5	6	25
Triglycerides	3	4	4	5	16
Heart Diseases	3	3	3	3	10
Unipolar Depression	3	4	3	4	32
Myocardial Infarction	3	4	5	6	15
Malignant neoplasm of prostate	3	4	3	4	18
Enthesitis-Related Arthritis	3	4	3	4	25
Behavior	3	3	3	4	13

## Data Availability

Source code for the ASQ platform and relevant analysis scripts can be found via https://github.com/mrcieu/epigraphdb-asq. Tutorial on programmatically accessing the ASQ platform can be found via this Jupyter notebook https://github.com/MRCIEU/epigraphdb-asq/blob/master/analysis/notebooks/programmatic-access.ipynb.
