# Supplementary material for: Triangulating evidence in health sciences with Annotated Semantic Queries
Source: Bioinformatics. Author manuscript; Available in PMC 2024 Sep 11. (PMC11377847; doi:10.1093/bioinformatics/btae519)
Supplement: Supplementary Materials [file EMS198302-supplement-Supplementary_Materials.pdf]

SUPPLEMENTARY MATERIALS FOR *Triangulating evidence in health sciences with Annotated Semantic Queries*

Yi Liu[1,*] and Tom R Gaunt[1,2,*]

[1] MRC Integrative Epidemiology Unit, Bristol Medical School, University of Bristol, Bristol, United Kingdom
[2] NIHR Bristol Biomedical Research Centre, University of Bristol, Bristol, United Kingdom
[*]Corresponding author: yi6240.liu@bristol.ac.uk, tom.gaunt@bristol.ac.uk

2024-08-16

S1 Glossary

Here we describe the various terms used in the manuscript and reference the main sections where these terms either first appear or are the topics for discussion.

- **Evidence triangulation** (Section 1) is the practice to obtain "more reliable answers" for research questions based on combining results from several approaches, and the answers will have more confidence if "the results of different approaches" reaches the same conclusion (Lawlor et al. [2017]). In the context of this research, our focus is on providing mechanisms both in terms of schematic design as well as software automation to combine various heterogenous evidence from a knowledge graph (EpiGraphDB) to assess the claims.

- **Semantic triple** (or *triple*; Section 1) is an atomic data entity in the form of `Subject PREDICATE Object` expression. A **claim triple** (Section 2) is then a specific triple derived from the input query text representing a single claim, e.g. "Obesity CAUSES Asthma", which the retrieved evidence are triangulated against in ASQ. An *evidence triple* is how a single **evidence item** (Section 2) from EpiGraphDB expressed, which we generally refer to as an evidence item rather than an evidence triple to avoid confusion with a claim triple.

- **Taxonomy** (Section 3.1) is the catalog of terms specific to a domain, i.e. the catalog of trait names of GWASes, the catalog of UMLS terms, and catalog of EFO terms are all separate taxonomies in their own domain, where a member of one taxonomy does not necessarily have corresponding counterparts in another taxonomy.

- **Entity** (Section 3.1) is a member of a taxonomy comprising an identifier and a label. *Claim entities* are the subject and object terms from the claim triple, and in ASQ they are denominated as SemRep / UMLS terms. *Evidence entities* are the source and target nodes of EpiGraphDB evidence triples under their own taxonomies and evidence groups. In this study we do not consider predicates (e.g. UMLS relationships "CAUSES", "TREATS", etc.) as entities and will refer to them always as predicates.

- **Entity harmonization** (Section 3.1) is the process of mapping various entities from different taxonomies with their close counterparts in an *ontology* (in our case this is the Experimental Factor Ontology, EFO).

- **Evidence harmonization** (Section 3.2 and Section 3.3) is the process of mapping various evidence item for the triangulation of the claim. Harmonization of an evidence item involves multiple dimension regarding the source nature and how it relates to the claim.

  - **Evidence group** (Section 3.2) is the categorization of an evidence item regarding the nature of its data source, i.e. whether it is a evidence from triple and literature sources, or from statistical association sources.
  - **Predicate group** (Section 3.3) is the categorization of predicates both in claim triples and in evidence items, i.e. whether there is a direction indication for the predicate or not, e.g. "TREATS" versus "ASSOCIATED_WITH".
  - **Evidence type** (Section 3.3) is the categorization of an evidence item regarding how it relates to the claim triple, i.e. whether it supports the claim.

- **Prioritisation scores** are scores which ASQ use to measure how relevant and strong the retrieved evidence is with respect to the query of interest.

  - **Mapping score** (Section S3, Eq 1) measures how the evidence relates to the query claim of interest in terms of semantic similarities by both the subject and object.
  - **Strength score** (Section S3, Eq 2a and Eq 3a) measures the strength of the evidence *per se* regardless of its relationship with the query claim.
  - **Evidence score** (Section S3, Eq 2b and Eq 3b) measures how the evidence relates to the query claim of interest in terms of its evidence strength, taking into account the semantic similarities.

S2 Technical details on entity harmonization

In ASQ we measure the proximity between two entities in the semantic space by the **semantic similarity** of their labels, which is calculated as the cosine similarity ($[0, 1]$) between the text embedding vectors of the labels. Specifically, ontology terms from the `(Efo)` nodes, UMLS terms from the `(LiteratureTerm)` nodes, and GWAS traits from the `(Gwas)` nodes are pre-encoded by ScispaCy (Neumann et al. [2019]) (`en_core_sci_lg-0.4.0`) into high-dimensional embedding vectors in an Elasticsearch vector store (Figure 1 left), which allows for fast retrieval of candidate entities via a k-nearest neighbor (kNN) search of the pre-computed vectors against the on-the-fly encoded vector of the query terms. On the one hand, entity representation via fast text embeddings is a naive approach on its own and candidate retrieval based on cosine similarity search can be highly sensitive to minor changes of the threshold, and on the other hand, sophisticated classification of entity relationships requires real-time inferencing between a large volume of candidate pairs using a dedicated classification model which is computationally resource-heavy. Thus for entity harmonization we implemented a two-stage approach that in the first stage the query terms are mapped to a handful of their close ontological representations in EFO, where simple semantic similarity measures are augmented by a dedicated ontology classification process (discussed below), and in the second stage greater number of evidence entities are retrieved for the corresponding EFO entities with kNN from the vector store. This enables robust and efficient retrieval of entities and evidence by ASQ.

The entity harmonization process starts with the retrieval of candidate EFO entities that semantically resemble the query terms, where ASQ attempts to select EFO entities that would qualify as either identical ontological representations of the query terms or as closely associated members in the hierarchy based on their **identity score** ($[0, +\infty)$) with the query terms. The identity score is produced by BlueBERT-EFO (Liu et al. [2023]) which we trained on the term mappings of EFO-EFO terms and GWAS-EFO terms to infer the distance (number of steps/nodes) between a query term and an EFO term in the ontology tree. An identity score of 0 suggests the two terms are equivalent in the ontology, whereas a score of 1 suggests that the term of interest can be considered as either a direct parent term or a direct descendant term of the reference ontology term (in practice this can be relaxed to 1.5 as the inference model produces a *regression* score rather than a *classification* score) and scores above 2 suggest greater distance between the two terms. In previous research on the performance of entity retrieval by various methods (Liu et al. [2023]) we showed that BlueBERT-EFO as a task-specific bespoke model is able to retrieve candidate terms that are closer to a term of interest in the semantic rankings, than naive embeddings from general purpose models (e.g. ScispaCy, BioSentVec (Chen et al. [2019]), etc.). The retrieval of EFO candidates is also augmented with a pre-filtering step to remove ontology candidate terms that are overly generic to mitigate scenarios where retrieved evidence entities in subsequent steps are less relevant to query terms due to these evidence entities being mapped to generic ontology terms (such as an ontology term “disease”). This is done via the pre-computed **information content** (**IC**) **score** of EFO terms using a scaled Sanchez Information Content (Sánchez and Batet [2012]) ($[0, 1]$) score where terms closer to an end node of the EFO tree have scores closer to 1 and terms closer to the origin have scores closer to 0. In both the interactive session and batch analysis mode, ASQ by default (identity score ≤ 1.5) seeks to select EFO candidates that would be either equivalent in the ontology to the query term, or a first-degree neighbor of it, as the basis for evidence identification, and in the interactive session users are able to further finetune the selection with the rest of the retrieved EFO candidates.

S3 Score metrics to measure and prioritize retrieved evidence

Here we discuss scores for the retrieved evidence in order to facilitate the assessment of individual evidence items and provide a simple way to compare between evidence items and groups. However as naive assessment metrics they should be used for simple comparisons and should not replace the actual investigation into specific evidence details.

The mapping score P_{mapping} ($[0, 1]$; Equation 1) of retrieved evidence measures the overall deviation in terms of semantic similarity (S) between the retrieved evidence entities and the original query claim terms, which is a product of semantic similarity scores of associated entities in the entity harmonization stage. A high score indicates that the retrieved evidence is of high semantic proximity to the query claim of interest, whereas a low score suggests that the semantic relevance of the retrieved entity to the claim is low and therefore the relevance of the evidence to the query should be discounted by the low semantic relevance. If multiple j EFO entities are identified for a query term, but these map to the same evidence entity, the route with the highest score value is chosen as the basis for mapping score calculation. In addition, for triple entities the query terms are added as *pseudo*-ontology entities as they share the same UMLS taxonomy.

$$P_{\text{mapping}} = \prod_{i} \max_{j} \left(S_{\text{query}\to\text{EFO}_j} \times S_{\text{EFO}_j\to\text{evidence}} \right), i \in [\text{subject}, \text{object}] \tag{1}$$

For *triple and literature* evidence, the strength of the evidence $P_{\text{T\&L.}}$ ($[1, +\infty)$; Equation 2a) is measured by the number of source literature items containing the semantic triple evidence. The baseline for the evidence score is 1 where the semantic triple is associated with 1 source literature article. Therefore the *evidence score* for *triple and literature* evidence $E_{\text{T\&L.}}$ ($[0, +\infty)$; Equation 2b) is a product of the evidence strength and mapping score, where in a typical scenario when there is an exact mapping (mapping score 1) of the involved entities and there is 1 source literature article the individual evidence item will have a baseline score of 1.

$$P_{\text{T\&L.}} = 1 + log_{10} N_{\text{literature}} \tag{2a}$$

$$E_{\text{T\&L.}} = P_{\text{mapping}} \times P_{\text{T\&L.}} \tag{2b}$$

For *association* evidence, the strength of the evidence $P_{\text{Assoc.}}$ ($[0, +\infty)$; Equation 3a) is measured with the standardized effect size of the statistical results where a unit absolute standardized effect size produces a score of 1 as the baseline. Similarly the evidence score for *association* evidence $E_{\text{Assoc.}}$ ($[0, +\infty)$; Equation 3b) is the product of the mapping status and the association evidence strength.

$$P_{\text{Assoc.}} = \max \left(0, 1 + \log_{10} \left| \frac{\beta}{\sigma} \right| \right) \tag{3a}$$

$$E_{\text{Assoc.}} = P_{\text{mapping}} \times P_{\text{Assoc.}} \tag{3b}$$

ASQ calculates the aggregate score and average score for each of the evidence groups and evidence types. Simple comparisons could be made (but should not be the substitute for further investigations) in cases such as between the supporting evidence group and reversal evidence group of a claim, as well as between two supporting evidence items. However comparisons between the supporting evidence group and the insufficient/additional evidence groups via quantitative measures are not appropriate as insufficient/additional evidence types by definition do not assess the query claim by metrics, and should instead be interpreted by the user based on their own knowledge. Similarly comparisons between the *triple and literature* group and the *association* group by metrics is not appropriate as they do not share a common measurement unit for their scores.

S4 Empirical analysis on retrieval of entities and evidence

Here we conduct an empirical analysis regarding the performance of various methods for querying EpiGraphDB, involving:

1. direct queries with the terms of interest to EpiGraphDB (*direct query* method), and

2. queries with terms of interest via ASQ with its semantic mappings between the terms of interest and the candidate entities, under various semantic thresholds (*ASQ* method).

This is to assess how semantic mappings as provided by ASQ can improve knowledge discovery, i.e. finding information relevant to a user's research interest at varying degrees of relevancy. Although our analysis here is specifically about ASQ and EpiGraphDB, similar conclusions and insights can be applied to a more general cases for using semantic mappings to improve the retrieval of entities and evidence from knowledge graphs involving heterogenous types of curated information.

Specifically, here we use all 275 unique query terms (*terms of interest*) from the extracted query subjects and objects in the medRxiv dataset described in Section 4.1 to query EpiGraphDB. For *direct query* method, the *terms of interest* are queried from EpiGraphDB API directly using exact text mapping with two meta nodes from EpiGraphDB – `(Gwas)` (GWAS traits which are main entities involved for association evidence) and `(LiteratureTerm)` (derived literature terms which are main entities for literature evidence). For *ASQ* method, the *terms of interest* are queried from ASQ API at different levels of semantic thresholds: 0.99, 0.95, 0.90, 0.85, 0.80, and 0.70 (current default setting for ASQ). The higher the semantic threshold the stricter the mapping will be and therefore fewer candidates will be retrieved, and a semantic threshold of 1 be return identical results to those from *direct query* method. Lower levels of thresholds will return entities and evidence less relevant to the query of interest in terms of semantic similarity, and ASQ will penalise those information by giving them low *entity mapping scores*.

For each of the retrieved entities under the two methods, we then additionally retrieve the corresponding evidence from EpiGraphDB based ASQ's schema of evidence harmonization (i.e. Table 2). We then analyse the retrieved evidence items both in terms of number of items as well as their strength with respect to the query terms.

As shown in Table S2, for 275 query terms in total the *direct query* method returns 170 corresponding entities from `literature_term` and 36 from `gwas`. These evidence terms are the those in EpiGraphDB that are identical to the query terms, i.e. for query terms such as "Mood Disorders", "Heart Diseases", etc. we are able to find their counterparts in the curated GWAS traits and literature terms in EpiGraphDB. As expected there are much lower matches from GWAS traits as they often describe more complex terms, e.g. UK Biobank questionnaire titles from which the GWASes are based on. ASQ method with a more tolerant threshold returns substantially more entities, and as shown in Table S2 there is still benefit even for a stringent threshold of 0.99, as direct matches would not be able to identify minor differences such as variations in spelling or expression (e.g. "Hypertensive disease" versus "Hypertension"), punctuation (e.g. "Parkinson Disease" versus "Parkinson's Disease"), etc.

Table S1: **Retrieval of EpiGraphDB entities using two query methods**
For each of the query terms, we query EpiGraphDB using the two query methods. For the *ASQ* method, different thresholds of semantic similarity is applied. The "both" column refers to entities that are both in the Gwas trait terms and literature terms.

`gwas`	`literature_term`	both	total	method
36	170	26	275	direct
49	173	39	275	asq_0.99
68	176	51	275	asq_0.95
108	201	87	275	asq_0.90
158	237	142	275	asq_0.85
193	257	182	275	asq_0.80
275	275	275	275	asq_default(0.7)

Table S2: **Examples of retrieved entities for ASQ method at 0.99 semantic threshold**

Query term	Matched terms
Mood Disorders	['Mood disorders']
Heart Diseases	['Heart failure', 'Coronary heart disease', 'Coronary heart disease', 'Coronary heart disease', 'Coronary heart disease']
Depressive disorder	['Mood disorders', 'Major Depressive Disorder', 'Major depressive disorder', 'Major depressive disorder', 'Major Depressive Disorder']
Hypertensive disease	['Hypertension']
Lupus Erythematosus, Systemic	['Systemic lupus erythematosus']

As shown in Table S3, as the retrieved entities are scarce from direct queries, the numbers of their corresponding evidence items are also low, and the ASQ method with lower threshold will retrieve substantially more evidence. Figure S1 and Figure S2 show the distribution of strength scores and evidence scores from the retrieved evidence for association evidence and literature triple evidence respectively. As the retrieved evidence are based on ASQ schema, it is expected that for strength scores they exceed the respective threshold for association evidence and triple evidence respectively. In addition, we would also expect the overall evidence scores to decrease at lower semantic thresholds as they are penalised for the evidence items where the associated entities have lower semantic similarities to the query terms. Nevertheless information with less semantic relevancy to the query do not necessarily mean they would not be of interest to the user, and ASQ has provided mechanisms for the user to do further investigation about those individual items. As mentioned in the main text, users are able to apply their own semantic threshold when performing their queries.

Table S3: **Retrieval of EpiGraphDB evidence using two query methods**
For the entities retrieved based on the two query methods (Table S2) we then retrieve the corresponding evidence, where the source and target of the evidence triple must both be members of the retrieved entities. The evidence items are categorised by the evidence type (association or literature evidence) and by evidence group (supporting, reversal, etc).

evidence_group	filter_type	Supporting	Reversal	Insufficient (directional)	Insufficient (non-directional)
assoc	direct	0	0	0	7
assoc	asq_0.99	1	0	0	7
assoc	asq_0.95	88	15	51	61
assoc	asq_0.90	160	35	184	434
assoc	asq_0.85	485	119	598	1565
assoc	asq_0.80	1000	208	1413	3314
assoc	asq_default(0.7)	3887	595	3976	8800
triple	direct	109	34	0	0
triple	asq_0.99	113	35	0	0
triple	asq_0.95	260	78	0	0
triple	asq_0.90	861	241	0	0
triple	asq_0.85	1688	404	0	0
triple	asq_0.80	2591	715	0	0
triple	asq_default(0.7)	3697	1168	0	0

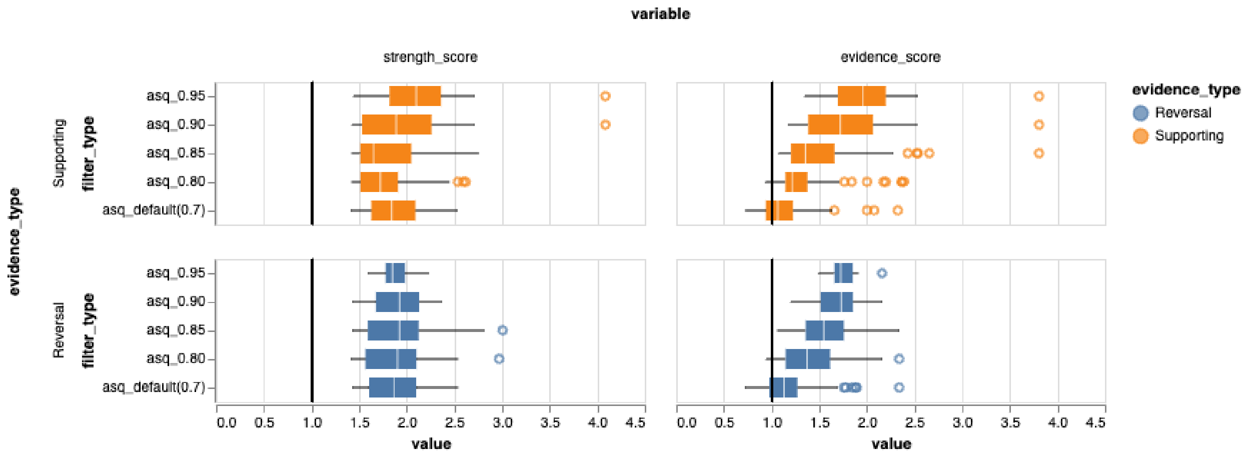

Figure S1: **Distribution of the retrieved association evidence under the two query methods**

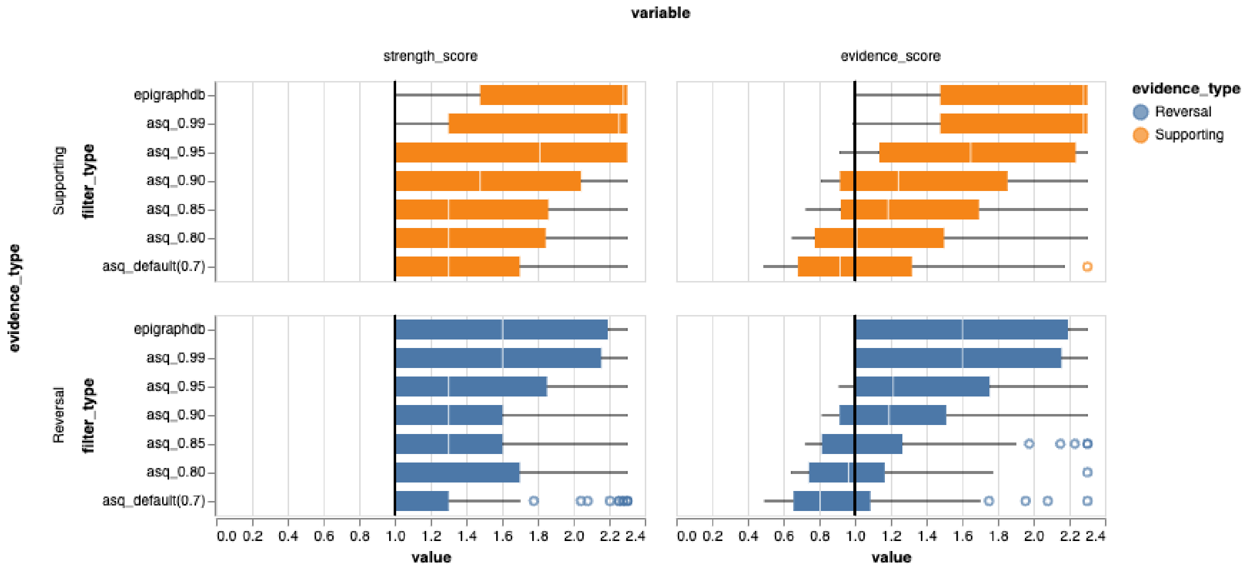

Figure S2: **Distribution of the retrieved literature triple evidence under the two query methods**

S5 Supplementary tables

Table S4: **EpiGraphDB knowledge on UMLS terms and relationships**
Summary counts of UMLS terms (top) and UMLS relationships (bottom) by their respective categories covered in this study. The UMLS terms and predicates are integrated as `(LiteratureTerm)` and `(LiteratureTriple)` knowledge in EpiGraphDB (version 1.0). Query text is parsed into a set of UMLS triples consisting of UMLS terms as subjects and objects, and UMLS relationships as predicates. In addition, the triple evidence retrieved also consists of UMLS triples as available from EpiGraphDB. **Top**: UMLS term (e.g. `C0424678` "Lean body mass") counts grouped by their semantic types, where the description of each semantic type is obtained from met. **Bottom**: UMLS semantic network relationships (uml [2009]) as covered in this study, where "predicate group" refers to the harmonized predicate group as discussed in Section 3.1.

UMLS terms		
**Semantic type**	**Description**	**Count**
gngm	Gene or Gnome	42,675
aapp	Amino Acid, Peptide, or Protein	34,713
orch	Organic Chemical	31,136
phsu	Pharmacologic Substance	15,192
dsyn	Disease or Syndrome	14,912
clnd	Clinical Drug	1,638
inch	Inorganic Chemical	1,584
hops	Hazardous or Poisonous Substance	1,555
enzy	Enzyme	1,036
horm	Hormone	823
clna	Clinical Attribute	342
chem	Chemical	9
UMLS predicates		
**Predicate**	**Predicate group**	**Count**
INTERACTS_WITH	Undirectional	1,272,318
COEXISTS_WITH	Undirectional	892,955
ASSOCIATED_WITH	Undirectional	302,338
CAUSES	Directional	200,813
TREATS	Directional	191,858
PRODUCES	Directional	179,281
AFFECTS	Directional	128,500

Table S5: **Knowledge triple and literature evidence**
Summary of triple and literature evidence counts by top triple groups. A triple evidence item takes the form of a UMLS triple e.g. "Obesity CAUSES Asthma" where the subject term *"Obesity"* (UMLS ID `C0028754`) and the object term *"Asthma"* (UMLS ID `C0004096`) both have type `dsyn` (Disease). For triple evidence items, we calculated the summary counts grouped by their predicate term, as well as the semantic types of subject and object entities, and then extracted the top 5 (by triple count) entries within each predicate group. We then calculated the number of literature evidence items associated with entry. Descriptions of the subject/object semantic types are available in Table S4.

Predicate group	Subject type	Object type	Triple count	Literature count
AFFECTS	aapp	dsyn	37,243	57,928
AFFECTS	gngm	dsyn	37,243	57,928
AFFECTS	dsyn	dsyn	29,167	58,753
AFFECTS	orch	dsyn	17,038	25,202
AFFECTS	phsu	dsyn	17,038	25,202
ASSOCIATED_WITH	aapp	dsyn	247,248	597,129
ASSOCIATED_WITH	gngm	dsyn	247,248	597,129
ASSOCIATED_WITH	phsu	dsyn	29,425	111,438
ASSOCIATED_WITH	enzy	dsyn	28,862	61,964
CAUSES	dsyn	dsyn	85,231	222,462
CAUSES	aapp	dsyn	49,178	125,153
CAUSES	gngm	dsyn	49,178	125,153
CAUSES	orch	dsyn	19,064	46,666
CAUSES	phsu	dsyn	19,064	71,138
COEXISTS_WITH	aapp	aapp	343,575	521,867
COEXISTS_WITH	gngm	gngm	343,575	521,867
COEXISTS_WITH	aapp	gngm	343,575	521,867
COEXISTS_WITH	gngm	aapp	343,575	521,867
COEXISTS_WITH	dsyn	dsyn	150,166	385,349
INTERACTS_WITH	gngm	gngm	694,873	1268,896
INTERACTS_WITH	gngm	aapp	682,155	1268,896
INTERACTS_WITH	aapp	gngm	675,239	1246,995
INTERACTS_WITH	aapp	aapp	662,521	1246,995
INTERACTS_WITH	aapp	phsu	126,849	221,742
PRODUCES	aapp	aapp	111,929	155,703
PRODUCES	aapp	gngm	111,929	155,703
PRODUCES	gngm	gngm	111,929	155,703
PRODUCES	gngm	aapp	111,929	155,703
PRODUCES	aapp	phsu	12,706	26,122
TREATS	phsu	dsyn	150,033	632,300
TREATS	orch	dsyn	82,263	274,589
TREATS	gngm	dsyn	20,354	119,075
TREATS	aapp	dsyn	20,354	119,075

Table S6: **Association evidence**

Summary of association evidence counts by association type and the category of the involved GWAS. An association evidence item is a quantitative relationship between two GWAS traits which comes from three EpiGraphDB relationship sources [MR_EVE_MR], [GEN_COR], [PRS]. For GEN_COR and PRS items we grouped them by the sub categories (e.g. ukb-b), and for MR_EVE_MR items due to the high pairwise density nature of the [MR_EVE_MR] relationship we grouped them by the broad categories (e.g. ukb) and extracted the top 5 entries to be succinct. Cells in column *GWAS categories* show the categories which the source and target GWAS-es belong to (e.g. for [ukb-a, ukb-b] it could be the source GWAS belongs to ukb-a and the target GWAS belongs to ukb-b, or the other way around). Descriptions on the GWAS categories are available from OpenGWAS documentation (Elsworth et al. [2020]).

Association type	GWAS categories	Evidence count
GEN_COR	[ukb-b, ukb-b]	453,752
GEN_COR	[ukb-a, ukb-b]	286,536
GEN_COR	[ukb-a, ukb-a]	180,536
GEN_COR	[ukb-b, ukb-d]	133,554
GEN_COR	[ukb-a, ukb-d]	84,266
GEN_COR	[ukb-d, ukb-d]	38,908
PRS	[ieu-a, ukb-a]	70,926
PRS	[ukb-b, ieu-a]	45,394
PRS	[ukb-a, ukb-a]	2,198
PRS	[ukb-b, ukb-a]	704
MR_EVE_MR	[ukb, ukb]	8,966,440
MR_EVE_MR	[prot, ukb]	5,028,904
MR_EVE_MR	[ubm, ukb]	3,833,948
MR_EVE_MR	[prot, prot]	3,109,406
MR_EVE_MR	[prot, ubm]	1,974,611

Table S7: **Notation conventions**
Notation for source biomedical entities and knowledge in EpiGraphDB. The graph database of EpiGraphDB models biomedical entities as `(nodes)` and their relationships as `[RELATIONSHIPS]`, where we refer to these sources in the context of the study using Neo4j Cypher syntax.

Graph elements	Label	Description
	EpiGraphDB nodes	
`(Efo)`	EFO	Experimental Factor Ontology (Malone et al. [2010]) which is widely used in categorizing GWAS traits.
`(LiteratureTerm)`	literature terms	UMLS Metathesaurus terms describing `(LiteratureTriple)`s curated in SemMedDB (Kilicoglu et al. [2012]). The subject and object entities of *triple and literature* evidence in ASQ.
`(LiteratureTriple)`	literature triple	Literature-derived semantic triples curated in SemMedDB. The semantic triple components of *triple and literature* evidence in ASQ.
`(Literature)`	source literature	PubMed (Canese and Weis [2013]) literature from which `(LiteratureTriple)`s are derived. The literature components of *triple and literature* evidence in ASQ.
`(Gwas)`	GWAS / GWAS traits	Traits of genome-wide association studies curated in OpenGWAS (Elsworth et al. [2020]). OpenGWAS curates the various GWAS set used in the association relationships below. The subject and object entities of *association* evidence in ASQ.
	EpiGraphDB relationships	
`[MR_EVE_MR]`	MR-EvE (Hemani et al. [2017])	Systematic analysis results of pairwise causal effects using Mendelian Randomization between two GWAS in MR-Base (Hemani et al. [2018]) and OpenGWAS (Elsworth et al. [2020]). Constituent source evidence for *association* evidence in ASQ.
`[PRS]`	PRS / PRS Atlas (Richardson et al. [2019])	Systematic analysis results of pairwise polygenic risk score associations between two GWAS in MR-Base and UKBiobank GWAS. Constituent source evidence for *association* evidence in ASQ.
`[GEN_COR]`	GEN_COR (Abbot et al. [2020])	Systematic analysis results of pairwise genetic correlations of the UKBiobank GWAS. Constituent source evidence for *association* evidence in ASQ.

Table S8: **Systematic analysis: parametric configuration**
Configurable parameters in ASQ in the processes of entity harmonization and evidence retrieval, and the specific values used in the systematic analysis results in Section 4.1. For conceptual discussions and technical details on the parameters please refer to Section 3.1 and Section S2 respectively. Further documentation on the ASQ platform can be found at https://asq.epigraphdb.org/docs.

Parameter	Description	Value
Semantic similarity threshold for ontology candidates	Semantic similarity between terms is calculated as the cosine similarity between two vectors of encoded terms via a text embedding model (ScispaCy Neumann et al. [2019]). The threshold is the primary metric in determining which ontology entities are retrieved as candidates in the entity harmonization.	0.7
Number of retrieved ontology candidates	Maximum number of candidates to retrieve from the vector store that are above the semantic similarity threshold.	10
Information content score threshold	The information content score measures the concreteness of a node in the ontology tree, with higher values associated with nodes towards the end of the branch. For example, for cancer related terms, the term "carcinoma" is roughly at 0.6. Candidates below the threshold will be removed, which is to mitigate scenarios where evidence entities retrieved in subsequent stages are of low relevancy to the query term due to them being mapped to a generic ontology term (e.g. "disease").	0.6
Identity score threshold	The identity score measures the relationship between the query term and the reference ontology in the ontology space, i.e. they are identical (closer to 0), a direct parent-descendant pair (closer to 1), relationship of further distance (greater than 2). The threshold determines which retrieved ontology *candidates* qualify as the associated ontology *entities* of the query term.	1.5
Semantic similarity threshold for evidence entities	Threshold for retrieving evidence (UMLS and GWAS trait) entities.	0.7
Number of retrieved evidence entities	Maximum number of retrieved evidence entities.	20
Statistical significance threshold	P-Value threshold to categorize association evidence items to the evidence types of "supporting", "reversal", and "insufficient". By default ASQ will seek to identify evidence items that quantitatively qualify at a sufficient statistically significant level but this behavior can be overridden by the user to other levels.	1e-2

Table S9: **Systematic analysis: summary statistics**
Entity count on claim triples, retrieved entities and retrieved evidence in the systematic analysis results. For the *claim triples* column the main value reports the number of terms (subjects and objects) in claim triples that are identified to contain associated entities and evidence in ASQ, and the value in parentheses reports the number of terms in the initial claim parsing sample. For the *EFO entities*, *UMLS entities*, and *trait entities* columns the main value reports the number of retrieved entities for the claim triples, and the value in parentheses reports the number of entities with semantic similarity scores above 0.85 (as a conventional threshold to signify entities that are similar to the claim terms). For the *T&L. evidence* (triple and literature evidence) and *Assoc. evidence* (association evidence) columns the main value reports the number of evidence items in the group across all evidence types, and the values in parentheses report the number of supporting evidence items and the number of supporting evidence items with evidence scores above 1.

Predicate	Claim triples	EFO entities	UMLS entities	Trait entities	T&L. evidence	Assoc. evidence
Directional predicates						
`AFFECTS`	85	344	7,984	1,432	1,955	7,735
	(2,487)	(288)	(3,806)	(679)	(375, 141)	(372, 196)
`CAUSES`	67	298	7,824	1,280	1,541	6,097
	(1,127)	(242)	(3,797)	(385)	(1,266, 482)	(412, 123)
`TREATS`	21	70	2,200	116	483	1,911
	(5,779)	(61)	(1,077)	(100)	(283, 143)	(83, 55)
Non-directional predicates						
`ASSOCIATED_WITH`	66	224	5,316	2,254	660	2,442
	(1,722)	(207)	(2,560)	(474)	(211, 73)	(3,106, 1,064)
`COEXISTS_WITH`	170	744	18,430	4,000	1,700	6,290
	(2,712)	(681)	(8,131)	(1,402)	(1,820, 736)	(2,401, 1,185)
`INTERACTS_WITH`	4	10	354	34	40	148
	(609)	(8)	(136)	(30)	(4, 1)	(46, 17)

Table S10: **Entity harmonization stage: distribution of query UMLS entities and EFO entities by UMLS semantic type**
Distribution of the identified query UMLS entities and harmonized EFO entities in the systematic analysis, ordered descendingly by EFO entity count to be in line with Figure 5 and Figure S3. The query UMLS entities are derived from parsing the MedRxiv abstracts from 2020-01-01 to 2021-12-31, and the EFO entities are retrieved from EpiGraphDB in the process of entity harmonization of the corresponding query UMLS entities.

Semantic type	Description	Query UMLS entity count	EFO entity count
`dsyn`	Disease or Syndrome	888	7,831
`mobd`	Mental or Behavioral Dysfunction	125	1,708
`neop`	Neoplastic Process	138	1,516
`sosy`	Sign or Symptom	124	562
`phsu`	Pharmacologic Substance	447	488
`bacs`	Biologically Active Substance	98	314
`patf`	Pathologic Function	171	303
`fndg`	Finding	509	266
`orch`	Organic Chemical	253	223
`orgf`	Organism Function	74	190
`aapp`	Amino Acid, Peptide, or Protein	487	152
`bpoc`	Body Part, Organ, or Organ Component	20	129
`horm`	Hormone	31	106
`orga`	Organism Attribute	31	77
`bhvr`	Behavior	2	76
`topp`	Therapeutic or Preventive Procedure	509	60
`ortf`	Organ or Tissue Function	74	47
`phsf`	Physiologic Function	44	42
`anab`	Anatomical Abnormality	9	30
`clna`	Clinical Attribute	24	30
`gngm`	Gene or Genome	504	20
`medd`	Medical Device	79	10
`hops`	Hazardous or Poisonous Substance	26	8
`cell`	Cell	49	8
`inpo`	Injury or Positioning	45	5

Table S11: **Evidence retrieval stage: summary statistics**
Summary of evidence scores and constituent scores for retrieved evidence in the systematic analysis, categorized by predicate group, predicate term, and evidence type. A score metric is reported as “aggregated value (average value)”, i.e. there are 372 retrieved association evidence items in the supporting evidence type with a predicate “AFFECTS”, with an aggregated score of 387.40 and an average score of 1.04 per item.

Predicate group	Predicate term	Evidence type	Item count	Strength score	Mapping score	Evidence score
		Association evidence				
**Directional**	**AFFECTS**	**Supporting**	372	676.04 (1.82)	212.94 (0.57)	387.40 (1.04)
		Reversal	343	634.07 (1.85)	206.75 (0.60)	382.35 (1.11)
		Insufficient	3,139	2,566.68 (0.82)	1,870.44 (0.60)	1,532.54 (0.49)
		Additional	289	322.96 (1.12)	166.20 (0.58)	183.41 (0.63)
	**CAUSES**	**Supporting**	412	719.92 (1.75)	204.93 (0.50)	361.96 (0.88)
		Reversal	399	700.80 (1.76)	199.85 (0.50)	354.40 (0.89)
		Insufficient	2,732	2,416.45 (0.88)	1,354.39 (0.50)	1,189.06 (0.44)
		Additional	1,111	2,127.96 (1.92)	499.12 (0.45)	928.82 (0.84)
	**TREATS**	**Supporting**	83	158.41 (1.91)	47.03 (0.57)	89.99 (1.08)
		Reversal	166	328.81 (1.98)	97.86 (0.59)	194.38 (1.17)
		Insufficient	111	100.73 (0.91)	57.31 (0.52)	52.16 (0.47)
		Additional	0	0.00 (N/A)	0.00 (N/A)	0.00 (N/A)
**Non-directional**	**ASSOCIATED_WITH**	**Supporting**	3,106	5,814.56 (1.87)	1,544.26 (0.50)	2,872.33 (0.92)
		Insufficient	5,272	4,570.48 (0.87)	2,839.05 (0.54)	2,441.49 (0.46)
	**COEXISTS_WITH**	**Supporting**	2,401	4,409.50 (1.84)	1,373.44 (0.57)	2,519.11 (1.05)
		Insufficient	7,315	6,202.52 (0.85)	4,261.65 (0.58)	3,623.38 (0.50)
	**INTERACTS_WITH**	**Supporting**	46	76.98 (1.67)	27.33 (0.59)	45.98 (1.00)
		Insufficient	174	157.41 (0.90)	102.48 (0.59)	92.98 (0.53)
		Triples & literature evidence				
**Directional**	**AFFECTS**	**Supporting**	375	497.90 (1.33)	273.09 (0.73)	367.50 (0.98)
		Reversal	291	385.62 (1.33)	211.45 (0.73)	285.21 (0.98)
	**CAUSES**	**Supporting**	1,266	1,779.61 (1.41)	875.66 (0.69)	1,244.00 (0.98)
		Reversal	954	1,279.71 (1.34)	632.01 (0.66)	847.10 (0.89)
	**TREATS**	**Supporting**	283	439.03 (1.55)	191.45 (0.68)	303.71 (1.07)
		Reversal	0	0.00 (N/A)	0.00 (N/A)	0.00 (N/A)
**Non-directional**	**ASSOCIATED_WITH**	**Supporting**	211	286.43 (1.36)	149.63 (0.71)	205.82 (0.98)
	**COEXISTS_WITH**	**Supporting**	1,820	2,638.37 (1.45)	1,269.99 (0.70)	1,857.41 (1.02)
	**INTERACTS_WITH**	Supporting	4	5.81 (1.45)	2.68 (0.67)	4.08 (1.02)

Table S12: **Systematic analysis results: top claim triples by retrieved evidence**

Top claim triples sorted by number of source claim abstract documents and the combined score between supporting triple and literature evidence as well as association evidence. For example, for claim "Coronavirus infections CAUSES Disease" there are 9 abstracts from MedRxiv articles associated with this claim, and EpiGraphDB-ASQ identifies 17 supporting triple and literature evidence ("T&L: S") items with an aggregated score of 21.19, and no supporting association evidence items ("Assoc: S"). In addition, for the reversal evidence to the claim (i.e. evidence supporting a claim "Disease CAUSES Coronavirus infections"), EpiGraphDB-ASQ identifies 8 reversal triple and literature evidence items ("T&L: R") with an aggregated score of 8.25, and 2 reversal association evidence items ("Assoc: R") with an aggregated score of 1.65. Reversal evidence is not applicable for "non-directional" claims without indication of direction in the predicate relationship. Interactive results on retrieved evidence for all claims in the systematic analysis are available on https://asq.epigraphdb.org/medrxiv-analysis.

Claim triple	Lit.	T&L: S	T&L: R	Assoc: S	Assoc: R
Directional predicates					
Coronavirus Infections CAUSES Disease	9	21.19 (17)	8.25 (8)	0.00 (0)	1.65 (2)
Blood Glucose AFFECTS Diabetes Mellitus, Non-Insulin-Dependent	2	3.89 (4)	2.49 (3)	9.55 (10)	9.55 (9)
Diabetes Mellitus, Non-Insulin-Dependent AFFECTS Parkinson Disease	2	4.61 (4)	5.77 (6)	1.97 (2)	0.96 (1)
Low Back Pain CAUSES Chronic pain	1	2.42 (4)	2.42 (4)	156.09 (222)	162.14 (225)
Valvular disease CAUSES Heart failure	1	74.78 (74)	41.35 (49)	32.68 (32)	25.39 (24)
Metabolic Diseases CAUSES Liver diseases	1	50.48 (57)	50.10 (58)	2.22 (2)	0.00 (0)
Heart Diseases CAUSES Pulmonary Hypertension	1	39.48 (35)	34.72 (39)	6.20 (8)	2.84 (3)
Myocardial Infarction CAUSES Acute myocardial infarction	1	11.67 (11)	10.76 (10)	31.11 (20)	29.84 (20)
Non-directional predicates					
Triglycerides COEXISTS_WITH Very low density lipoprotein	2	42.05 (47)	N/A	60.40 (54)	N/A
Depressive disorder COEXISTS_WITH Parkinson Disease	2	25.17 (24)	N/A	6.09 (5)	N/A
Hepatic impairment COEXISTS_WITH Disease	2	27.93 (27)	N/A	0.93 (1)	N/A
Chronic disease COEXISTS_WITH Obesity	2	17.80 (13)	N/A	1.04 (1)	N/A
Disease COEXISTS_WITH Diabetes	2	14.04 (11)	N/A	2.23 (3)	N/A
Fatigue ASSOCIATED_WITH Disease	2	0.80 (1)	N/A	15.14 (21)	N/A
Sleep ASSOCIATED_WITH Alzheimer's Disease	2	2.29 (3)	N/A	11.18 (8)	N/A
Malignant Neoplasms COEXISTS_WITH Disease	2	4.90 (5)	N/A	6.17 (6)	N/A

S6 Supplementary figures

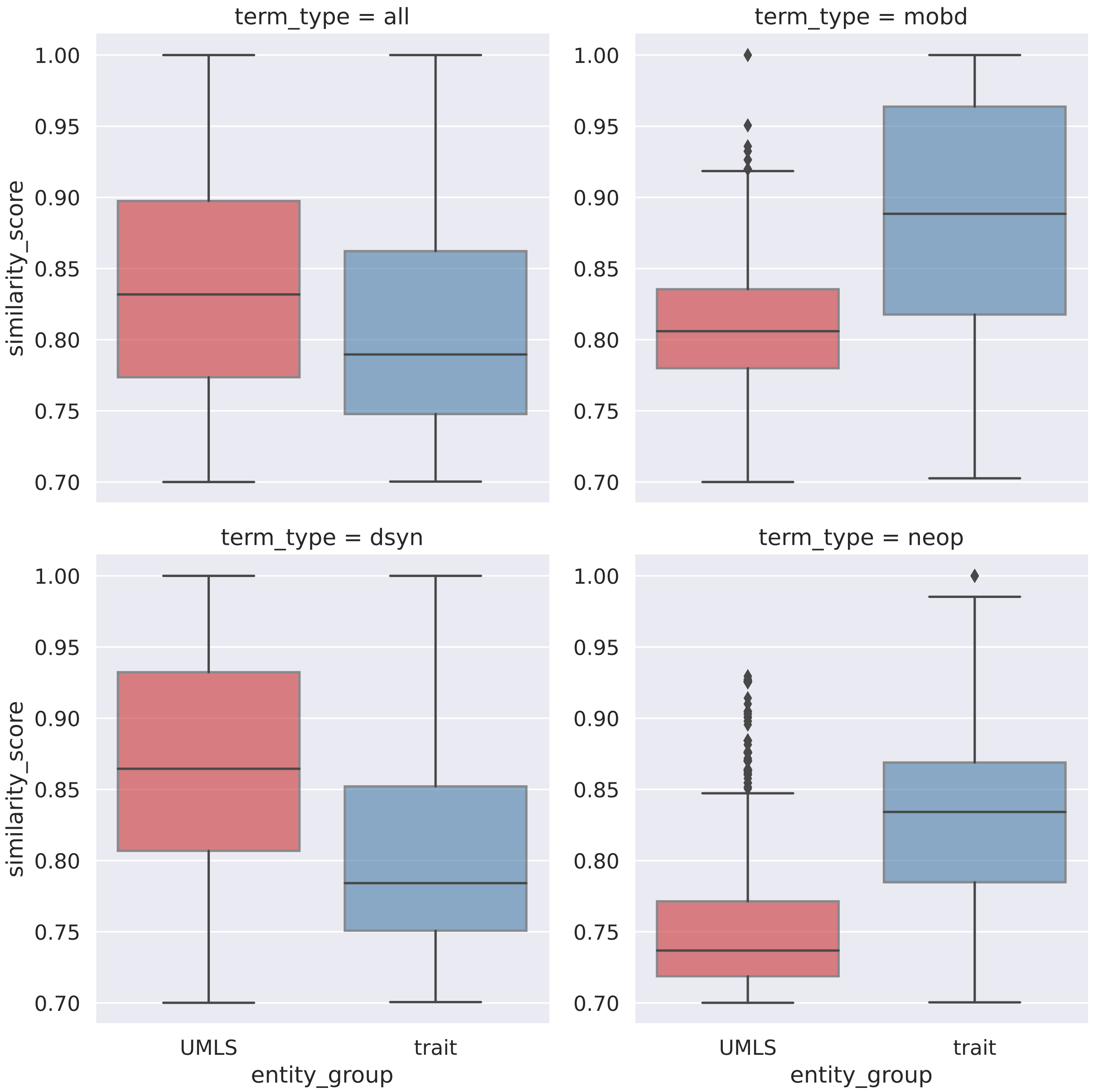

Figure S3: **Entity harmonization: distribution of score metrics of retrieved UMLS and trait entities**
Distribution of semantic similarity scores as metrics used for retrieved UMLS and trait entities in the process of mapping with EFO entities, categorized by the semantic type of the initial query UMLS terms where the EFO entities are mapped to. This figure reports distributions in the top 3 semantic type groups by entity count (Table S10 reports entity counts of all semantic types).

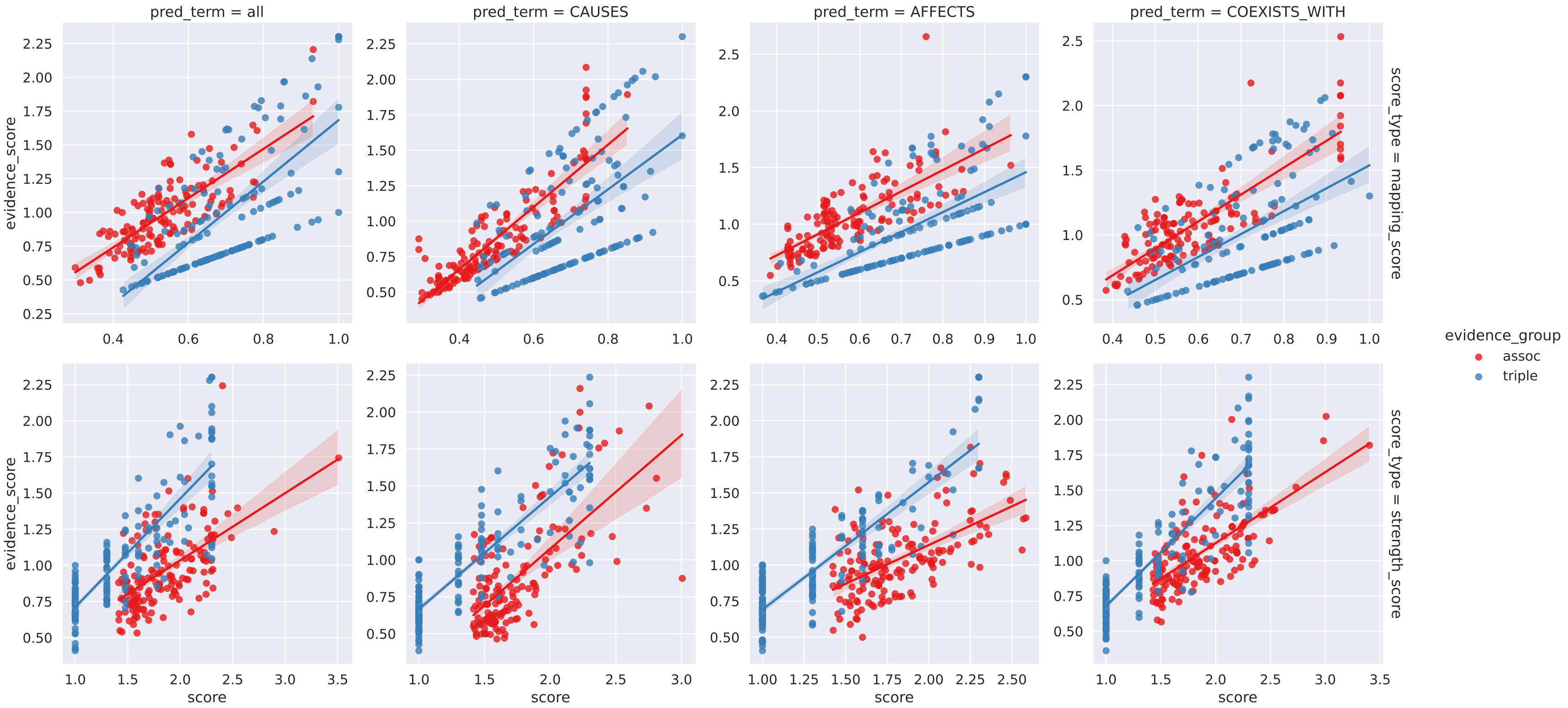

Figure S4: **Evidence retrieval stage: relationship between evidence scores with its constituent scores (supporting evidence)**
The scatter plots report the relationship between evidence scores and mappings scores (**top**), as well as between evidence scores and strength scores (**bottom**). Point colors correspond to evidence groups of triple and literature group ("triple") and association group ("assoc").

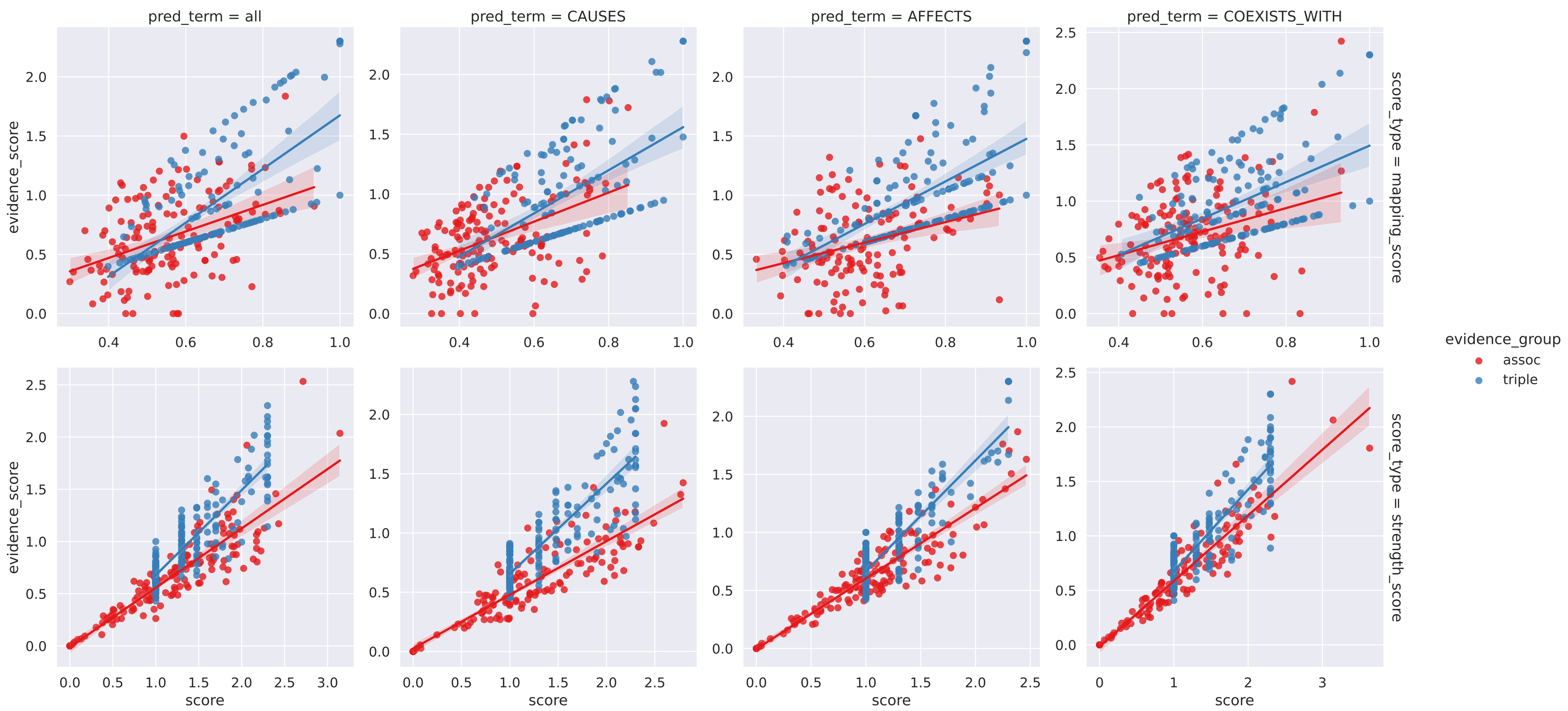

Figure S5: **Evidence retrieval: relationship between evidence score with its constituent scores (all evidence types)**
The scatter plots report the relationship between evidence scores and mappings scores (**top**), as well as between evidence scores and strength scores (**bottom**). Point colors correspond to evidence groups of triple and literature group ("triple") and association group ("assoc").

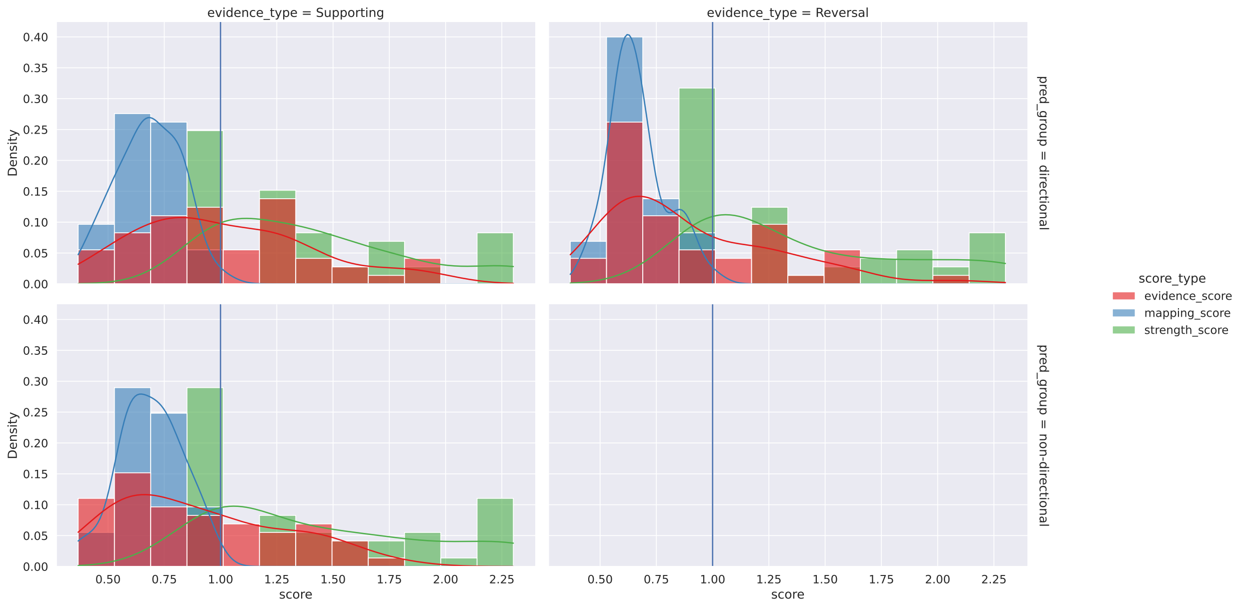

Figure S6: **Evidence retrieval stage: distribution of evidence scores and constituent scores (triple and literature evidence group)**
Distribution of evidence scores and its constituent scores (entity mapping scores and evidence strength scores), for all evidence types (by columns) in the triple and literature evidence group and by predicate groups (by rows).

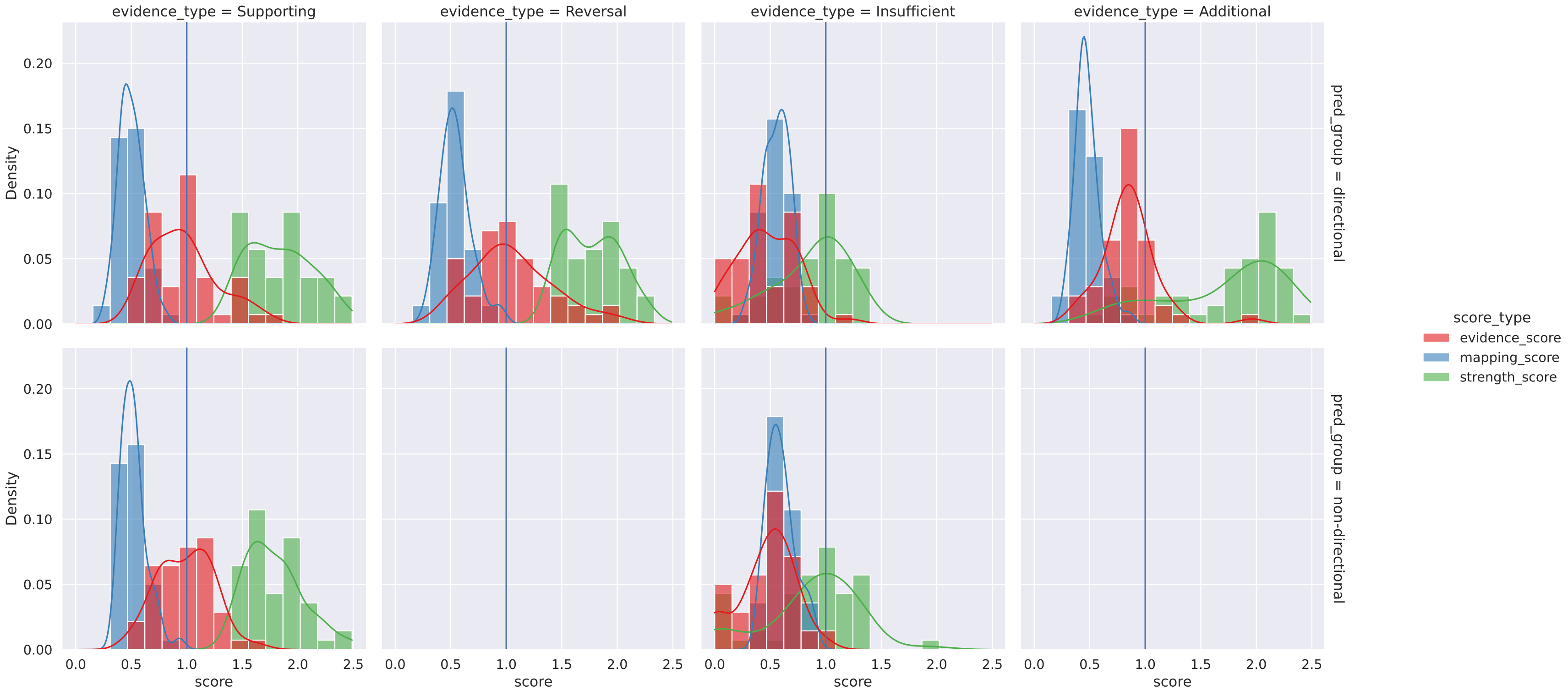

Figure S7: **Evidence retrieval stage: distribution of evidence scores and constituent scores (associations evidence group)**
Distribution of evidence scores and its constituent scores (entity mapping scores and evidence strength scores), for all evidence types (by columns) in the association evidence group and by predicate groups (by rows).

References

Semantic Types & Groups, MetaMap Online Documentation.

UMLS Reference Manual, chapter Semantic Network. National Library of Medicine, 2009.

L. Abbot, B. Neale, and D. Palmer. Genetic correlation between traits and disorders in the UK biobank. Technical report, Neale Lab, Analytical and Translation Genetics Unit, 2020.

K. Canese and S. Weis. Pubmed: the bibliographic database. *The NCBI handbook*, 2(1), 2013.

Q. Chen, Y. Peng, and Z. Lu. Biosentvec: creating sentence embeddings for biomedical texts. In *2019 IEEE International Conference on Healthcare Informatics (ICHI)*, pages 1–5. IEEE, 2019.

B. Elsworth, M. Lyon, T. Alexander, Y. Liu, P. Matthews, J. Hallett, P. Bates, T. Palmer, V. Haberland, G. D. Smith, J. Zheng, P. Haycock, T. R. Gaunt, and G. Hemani. The MRC IEU OpenGWAS data infrastructure. *bioRxiv*, 2020. doi: 10.1101/2020.08.10.244293.

G. Hemani, J. Bowden, P. Haycock, J. Zheng, O. Davis, P. Flach, T. Gaunt, and G. D. Smith. Automating mendelian randomization through machine learning to construct a putative causal map of the human phenome. *bioRxiv*, 2017. doi: 10.1101/173682.

G. Hemani, J. Zheng, B. Elsworth, K. H. Wade, V. Haberland, D. Baird, C. Laurin, S. Burgess, J. Bowden, R. Langdon, V. Y. Tan, J. Yarmolinsky, H. A. Shihab, N. J. Timpson, D. M. Evans, C. Relton, R. M. Martin, G. Davey Smith, T. R. Gaunt, and P. C. Haycock. The mr-base platform supports systematic causal inference across the human phenome. *eLife*, 7:e34408, may 2018. ISSN 2050-084X. doi: 10.7554/eLife.34408.

H. Kilicoglu, D. Shin, M. Fiszman, G. Rosemblat, and T. C. Rindflesch. Semmeddb: a pubmed-scale repository of biomedical semantic predications. *Bioinformatics*, 28(23):3158–3160, 2012.

D. A. Lawlor, K. Tilling, and G. Davey Smith. Triangulation in aetiological epidemiology. *International Journal of Epidemiology*, 45(6):1866–1886, 01 2017. ISSN 0300-5771. doi: 10.1093/ije/dyw314.

Y. Liu, B. L. Elsworth, and T. R. Gaunt. Using language models and ontology topology to perform semantic mapping of traits between biomedical datasets. *Bioinformatics*, 39(4):btad169, 04 2023. ISSN 1367-4811. doi: 10.1093/bioinformatics/btad169.

J. Malone, E. Holloway, T. Adamusiak, M. Kapushesky, J. Zheng, N. Kolesnikov, A. Zhukova, A. Brazma, and H. Parkinson. Modeling sample variables with an experimental factor ontology. *Bioinformatics*, 26(8):1112–1118, 2010.

M. Neumann, D. King, I. Beltagy, and W. Ammar. ScispaCy: Fast and Robust Models for Biomedical Natural Language Processing. In *Proceedings of the 18th BioNLP Workshop and Shared Task*, pages 319–327, Florence, Italy, Aug. 2019. Association for Computational Linguistics. doi: 10.18653/v1/W19-5034.

T. G. Richardson, S. Harrison, G. Hemani, and G. D. Smith. An atlas of polygenic risk score associations to highlight putative causal relationships across the human phenome. *Elife*, 8:e43657, 2019.

D. Sánchez and M. Batet. A new model to compute the information content of concepts from taxonomic knowledge. *Int. J. Semantic Web Inf. Syst.*, 8:34–50, 2012.
